# Pro-angiognetic and pro-osteogenic effects of human umbilical cord mesenchymal stem cell-derived exosomal miR-21-5p in osteonecrosis of the femoral head

**DOI:** 10.1038/s41420-022-00971-0

**Published:** 2022-04-25

**Authors:** Shanhong Fang, Zhaoliang Liu, Songye Wu, Xinjie Chen, Mengqiang You, Yongfeng Li, Fuhui Yang, Shuhuan Zhang, Yiqun Lai, Peiyao Liu, Weijiawen Jiang, Peng Chen

**Affiliations:** 1 Department of Orthopaedics, Fujian Orthopaedics Research Institute, The First Affiliated Hospital of Fujian Medical University, 350005 Fuzhou, P. R. China; 2 Department of Plastic Surgery, Research Institute of Plastic and Aesthetic Surgery, The First Affiliated Hospital of Fujian Medical University, 350005 Fuzhou, P. R. China; 3 Fujian Medical University, 350122 Fuzhou, P. R. China

**Keywords:** Diseases, Cell biology

## Abstract

Mesenchymal stem cell (MSC)-derived exosomes (Exos) enhanced new bone formation, coupled with positive effects on osteogenesis and angiogenesis. This study aims to define the role of microRNA (miR)-21-5p delivered by human umbilical MSC-derived Exos (hucMSC-Exos) in the osteonecrosis of the femoral head (ONFH). We first validated that miR-21-5p expression was downregulated in the cartilage tissues of ONFH patients. Besides, hucMSCs delivered miR-21-5p to hFOB1.19 cells and human umbilical vein endothelial cells (HUVECs) through the secreted Exos. Loss- and gain-of-function approaches were performed to clarify the effects of Exo-miR-21-5p, SOX5, and EZH2 on HUVEC angiogenesis and hFOB1.19 cell osteogenesis. It was established that Exo-miR-21-5p augments HUVEC angiogenesis and hFOB1.19 cell osteogenesis in vitro, as reflected by elevated alkaline phosphatase (ALP) activity and calcium deposition, and increased the expression of osteogenesis-related markers OCN, Runx2 and Collagen I. Mechanistically, miR-21-5p targeted SOX5 and negatively regulated its expression, while SOX5 subsequently promoted the transcription of EZH2. Ectopically expressed SOX5 or EZH2 could counterweigh the effect of Exo-miR-21-5p. Further, hucMSC-Exos containing miR-21-5p repressed the expression of SOX5 and EZH2 and augmented angiogenesis and osteogenesis in vivo. Altogether, our study uncovered the role of miR-21-5p shuttled by hucMSC-Exos, in promoting angiogenesis and osteogenesis, which may be a potential therapeutic target for ONFH.

## Introduction

Osteonecrosis of the femoral head (ONFH) is a disabling disease influencing a young population, which is also regarded as a major cause of total hip arthroplasty in this population [1]. The therapeutic protocol of mesenchymal stem cell (MSC) transplantation holds great potential when instilled during the early stage of ONFH, however its highly susceptible nature and interaction with the inhibited osteogenic differentiation and migration of the transplanted MSCs associated with pathological bone tissues persists as a challenge [2]. The damage of endothelial cells and suppressed osteogenesis serve as trivial hallmarks of glucocorticoid-triggered ONFH [3]. Improvement in angiogenesis and osteogenesis has been proposed as conducive protocols for bone regeneration, thus eliciting potential as treatment options to prevent or treat ONFH [2]. Existing research has highlighted the potential role of human umbilical cord MSC-derived exosomes (hucMSC-Exos) in the ONFH by facilitating angiogenesis in the necrotic bone tissues of rats [4]. Identifying the underlying mechanism of angiogenesis and osteogenesis may provide a scientific basis for clinical treatment for ONFH.

Exos are small single-membraned vesicles approximately 30–200 nm in diameter, enriched in plenty of proteins, lipids, nucleic acids, and glycoconjugates [5]. Published research supports the therapeutic functionality of MSC-derived Exos on ONFH [6]. Exos can serve as a carrier of microRNAs (miRNAs) and augment the proliferative capacity and differentiation of osteoblasts [7, 8]. Moreover, miRNAs, a class of non-coding RNAs, elicit vital functionality in the pathogenesis of bone diseases, including ONFH [9, 10]. Intriguingly, the enrichment of miR-21-5p in bone MSC-Exos promotes angiogenesis and fibroblast function, improving wound healing [11]. Meanwhile, hucMSC-Exos containing miR-21 can radically inhibit osteocyte apoptosis in glucocorticoid-induced ONFH in rats [12].

Our bioinformatics analysis predicted that SRY-box transcription factor 5 (SOX5) is a putative target of miR-21-5p. SOX5 is a transcription factor in preadipocytes [13], and essentially serves as a vital indicator for prechondrocyte differentiation into chondroblasts and then chondrocytes [14]. SOX5 can positively modulate the expression of enhancer of zeste homologue 2 (EZH2) in the context of breast cancer [15]. As a histone H3 lysine 27 methyltransferase, EZH2 stimulates tumorigenesis in multiple cancers [16]. Downregulation of EZH2 contributes to the prevention of fibrosis and promotion of normal angiogenesis in scleroderma [17].

Therefore, we hypothesized that the transfer of miR-21-5p via hucMSC-Exos might affect angiogenesis and osteogenesis during ONFH, and thus we sought to test this hypothesis in vitro and in vivo.

## Results

### hucMSCs augment human umbilical vein endothelial cell (HUVEC) angiogenesis and hFOB1.19 cell osteogenesis by secreting Exos

To identify the effect of hucMSC-Exos on angiogenesis and osteogenesis, we initially isolated hucMSCs from human umbilical cords. Under microscopy, the isolated cells exhibited a spindle-shaped fibroblast-like morphology (Supplementary Fig. 1A). The differentiation of cells in the osteogenic and adipogenic mediums into osteoblasts adipocytes, or chondrocytes was evident as determined with Alizarin Red, oil red O, and Alcian blue staining, respectively (Supplementary Fig. 1C). The results of flow cytometry analysis showed that the isolated cells exhibited highly expressed MSC positive markers CD29, CD44, CD73, and CD90, without expressing negative markers CD34 and CD45 (Supplementary Fig. 1B). These results were indicative of successful hucMSC isolation.

Next, we co-cultured the isolated hucMSCs with the hFOB1.19 cells or co-cultured the hucMSCs treated with 10 μM GW4869 (Exo release inhibitor) with hFOB1.19 cells. A combination of Alizarin red S and alkaline phosphatase (ALP) staining demonstrated that the hucMSC treatment led to increased ALP activity and calcium deposition, while further GW4869 treatment reduced the ALP activity and calcium deposition (Supplementary Fig. 1D, E).

Next, we conducted immunoblotting to determine the protein expression of osteogenesis-related markers OCN, Runx2, and Collagen I in hFOB1.19 cells, the results of which revealed increased OCN, Runx2, and Collagen I protein expression in the presence of hucMSCs, while their protein levels had decreased due to subsequent GW4869 treatment (Supplementary Fig. 1F).

Subsequently, we co-cultured hucMSCs with HUVECs. The Transwell and capillary-like tube formation assays showed an increased number of migrated cells and elevated tube formation ability in hucMSCs-co-cultured HUVECs, whose effects were reversed by further GW4869 treatment (Supplementary Fig. 1G, H).

In summary, hucMSCs augment HUVEC angiogenesis and hFOB1.19 cell osteogenesis by secretion of Exos.

### hucMSC-Exos containing miR-21-5p augment HUVEC angiogenesis and hFOB1.19 cell osteogenesis

An existing study proposed that miR-21 is enriched in hucMSC-Exos [18]. Meanwhile, the overexpression of miR-21 could inhibit osteoblast apoptosis, relieve ONFH-induced by glucocorticoids, and augment angiogenesis in vitro [12, 19]. Based on RT-qPCR data, decreased expression of miR-21-5p was revealed in the cartilage tissues of ONFH patients (Supplementary Fig. 2A).

Next, we extracted Exos from the supernatant of hucMSCs, which exhibited basically consistent round- or elliptical-shaped membranous vesicles under a TEM (Supplementary Fig. 2B). The dynamic light scattering analysis results showed that the diameter of the extracted sample predominantly ranged from 30 to 120 nm (Supplementary Fig. 2C). Subsequent immunoblotting results identified positive expression for Exo markers CD63, CD81, and TSG101, and negative expression of the endoplasmic reticulum marker protein Calnexin in isolated Exos (Supplementary Fig. 2D). These results were indicative of the successful extraction of Exos.

Next, we labeled the hucMSC-Exos with PKH67 (Green) for co-culture with hFOB1.19 cells and HUVECs for 24 h. Under a confocal fluorescence microscope, we observed the ability of hFOB1.19 cells and HUVECs to internalize the hucMSC-Exos (Supplementary Fig. 2E). We continued to co-culture miR-21-5p-Cy3-transfected hucMSCs with pCDNA3.1-GFP-transfected hFOB1.19 cells and HUVECs. Red fluorescence was observed from the hFOB1.19 cells and HUVECs (Supplementary Fig. 2F). Further, a notably increased expression of miR-21-5p was identified in hucMSC-Exos in response to miR-21-5p mimic (Supplementary Fig. 2G). Exo-mimic-NC treatment increased the expression of miR-21-5p in hFOB1.19 cells and HUVECs, while the Exo-miR-21-5p-mimic treatment led to a further increase, relative to the Exo-mimic-NC (Supplementary Fig. 2H). Together, hucMSCs delivered miR-21-5p to hFOB1.19 cells and HUVECs through the secreted Exos.

The ALP and alizarin red S staining results showed that the Exo-mimic-NC treatment induced an increase in ALP activity and calcium deposition while the miR-21-5p overexpression in Exos also notably increased ALP activity and calcium deposition (Supplementary Fig. 2I, J). Consistently, elevated protein expression of OCN, Runx2 and Collagen I in response to Exo-mimic-NC, and miR-21-5p overexpression in Exos further increased their levels (Supplementary Fig. 2K). Transwell and capillary-like tube formation assays displayed an increased number of migrated cells and tube formation ability in response to Exo-mimic-NC, accompanied by increased total network area, total length, and the number of branch points, and further increases were observed by Exo-miR-21-5p-mimic treatment relative to the Exo-mimic-NC (Supplementary Fig. 2L, M).

Collectively, hucMSC-Exos augment HUVEC angiogenesis and hFOB1.19 cell osteogenesis by delivering miR-21-5p.

### miR-21-5p targets SOX5

**Fig. 1:**
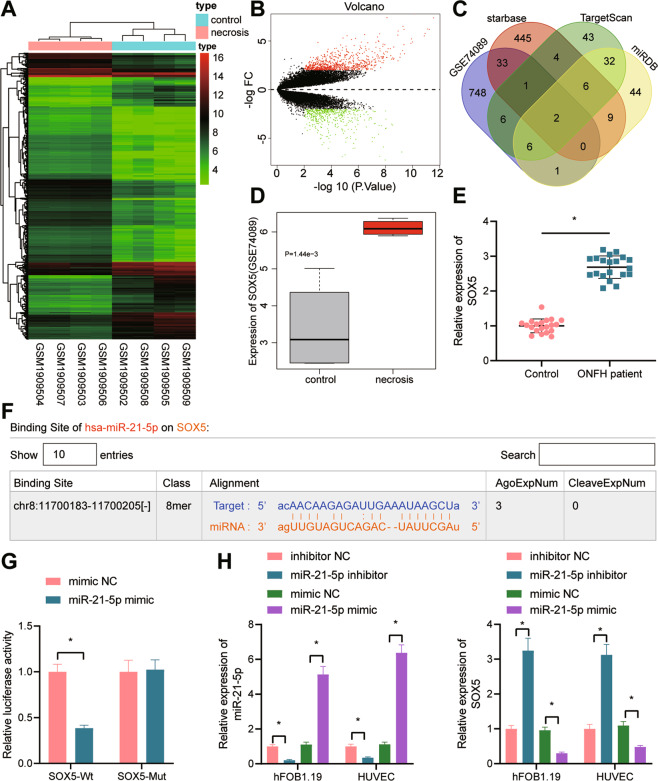
miR-21-5p targets SOX5 and negatively regulates its expression. **A** Heatmap of significantly differently expressed genes in ONFH samples of the GSE74089 dataset. Each row represents a differentially expressed gene, while each column represents a sample; **B** volcanic map of significantly differentially expressed genes in ONFH samples of GSE74089 dataset. Red represents upregulated genes, while green represents down-regulated genes, |logFC | > 2, *p* < 0.01; **C** the Venn diagram of intersection among significantly upregulated genes from the GSE74089 dataset, the top 500 target genes by miR-21-5p predicted by the starBase database and the top 100 target genes predicted by the TargetScan and miRDB databases; **D** SOX5 expression predicted in the GSE74089 dataset. Red box indicates ONFH samples, while gray box indicates normal samples; **E** SOX5 mRNA expression in the cartilage tissues of patients with ONFH (*n* = 20) and patients with femoral neck fracture (normal control, *n* = 20) determined with RT-qPCR; **F** the binding site of miR-21-5p and SOX5 predicted with TargetScan; **G** the targeting relationship between miR-21-5p and SOX5 verified with dual-luciferase reporter gene assay; **H** the expression of miR-21-5p and SOX5 in hFOB1.19 cells and HUVECs determined with RT-qPCR. Cell experiment was repeated three times independently. *p < 0.05.

To explore the downstream regulatory mechanism of miR-21-5p, we obtained the GSE74089 dataset of gene expression profiling of hip cartilage with ONFH, followed by differential analysis and identification of 797 significantly upregulated and 491 significantly downregulated genes (Fig. 1A, B). Next, we intersected the significantly upregulated genes from the GSE74089 dataset with the top 500 target genes by miR-21-5p predicted by the starBase database and the top 100 predicted by the TargetScan and miRDB databases. Only two intersection genes were identified: SOX5 and PLEKHA1 (Fig. 1C), of which SOX5 was more significantly upregulated in the ONFH samples of the GSE74089 dataset (Fig. 1D). Therefore, we speculate that miR-21-5p may target SOX5 to participate in ONFH.

Subsequent RT-qPCR data validated the increased expression of SOX5 in the cartilage tissues of patients with ONFH (Fig. 1E). Next, the miR-21-5p and SOX5 binding sites were predicted by the TargetScan database (Fig. 1F). Further, the dual-luciferase reporter gene assay showed that compared to the mimic-NC, the luciferase activity was decreased in response to co-transfection of miR-21-5p mimic with SOX5-WT, while no significant difference was evident in the luciferase activity in response to co-transfection with SOX5-MUT (Fig. 1G), which indicated the ability of miR-21-5p to specifically target SOX5 3’UTR.

We further modulated the expression of miR-21-5p in hFOB1.19 cells and HUVECs and found that SOX5 mRNA expression was reduced notably in response to miR-21-5p mimic, while its expression was markedly increased in response to miR-21-5p inhibitor (Fig. 1H).

Coherently, miR-21-5p targets SOX5 and inhibits the SOX5 expression in hFOB1.19 cells and HUVECs.

### SOX5 promotes the transcription of EZH2

**Fig. 2:**
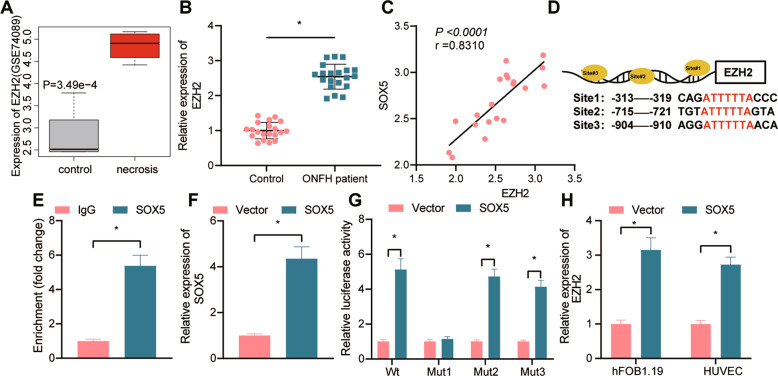
SOX5 upregulates the expression of EZH2. **A** The expression of EZH2 predicted in the GSE74089 dataset. Red box represents ONFH samples, and gray box represents normal samples, *p* < 0.05; **B** EZH2 mRNA expression in the cartilage tissues of patients with ONFH (*n* = 20) and patients with femoral neck fracture (normal control, *n* = 20) determined with RT-qPCR; **C** Pearson’s correlation coefficient of the correlation between the mRNA expression of SOX5 and EZH2 in ONFH samples; **D** the binding site between SOX5 and EZH2 promoter region as predicted with JASPAR CORE database; **E** ChIP assay of SOX5 enrichment in the EZH2 promoter region in HEK293T cells; **F** SOX5 mRNA expression in the HEK293T cells determined with RT-qPCR; **G** Targeting relationship between SOX5 and EZH2 validated with the dual-luciferase reporter gene assay; **H** EZH2 mRNA expression in hFOB1.19 cells and HUVECs assessed with RT-qPCR. Cell experiment was repeated three times independently. *p < 0.05.

SOX5 has been reported to increase the expression of EZH2 at the mRNA and protein levels, while the knock-down of SOX5 reduces the expression of EZH2 [15]. The downregulated expression of EZH2 induces angiogenesis and osteogenesis [17, 20]. To further explore the relationship between SOX5 and EZH2 in ONFH, we initially analyzed the GSE74089 dataset and found that EZH2 was notably upregulated in the ONFH samples (Fig. 2A). RT-qPCR data revealed that the expression of EZH2 was elevated in the cartilage tissues of patients with ONFH, accompanied by a positive correlation between SOX5 and EZH2 as reflected in Pearson’s correlation coefficient (Fig. 2B, C). Next, the JASPAR CORE database analysis suggested that the top three binding sites of SOX5 and EZH2 promoter region were as follows: site 1: −313 to −319, site 2: −715 to −721, site 3: −904 to −910 (Fig. 2D).

As shown by ChIP assay, SOX5 was enriched in the EZH2 promoter region (Fig. 2E). To validate the specific binding site, we constructed the promoter WT (full-length EZH2 promoter sequence), promoter MUT-site 1 (mutant site 1), promoter MUT-site 2 (mutant site 2), and promoter MUT-site 3 (mutant site 3) luciferase reporter plasmids, which demonstrated that the overexpression of SOX5 notably enhanced the luciferase activity in the promoter WT, promoter MUT-site 2 and promoter MUT-site 3 groups; however, no significant alteration was witnessed in the promoter MUT-site 1 group, thereby indicating that the binding site of SOX5 and the EZH2 promoter region was site 1 (Fig. 2F, G). To validate the regulation of SOX5 on EZH2, we overexpressed SOX5 in the hFOB1.19 cells and HUVECs. RT-qPCR results revealed that the overexpression of SOX5 notably increased the mRNA level of EZH2, which suggested that SOX5 promoted the transcription of EZH2 (Fig. 2H).

Overall, SOX5 could bind to the EZH2 promoter to enhance its transcription and augment its expression in hFOB1.19 cells and HUVECs.

### SOX5 suppresses HUVEC angiogenesis and hFOB1.19 cell osteogenesis by upregulating EZH2 expression

**Fig. 3:**
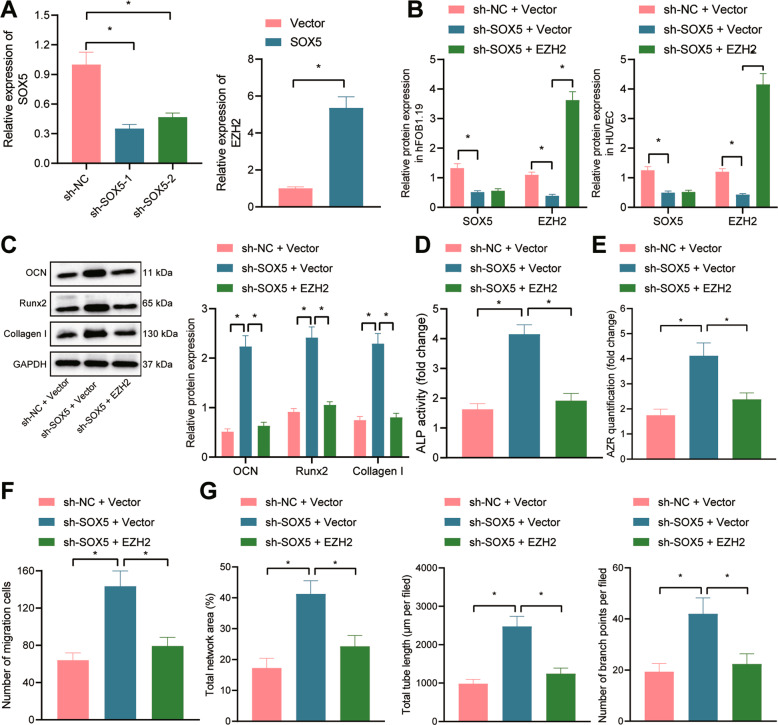
SOX5 inhibits HUVEC angiogenesis and hFOB1.19 cell osteogenesis by promoting EZH2 expression. **A** The transduction efficiency of SOX5 and EZH2 in cells validated with RT-qPCR; **B** immunoblotting analysis of the expression of SOX5 and EZH2 cells; **C** immunoblotting analysis of the expression of osteogenesis-related markers (OCN, Runx2, and Collagen I) in hFOB1.19 cells; **D** quantitative analysis of ALP activity of hFOB1.19 cells; **E** quantitative analysis of calcium deposition of hFOB1.19 cells by alizarin red S staining; **F** the migration of HUVECs determined with Transwell assay; **G** the tube-forming ability of HUVECs with total network area, total length, and the number of branch points determined with capillary-like tube formation assay. Cell experiment was repeated three times independently. *p < 0.05.

To explore the effect of SOX5 in the regulation of EZH2 on angiogenesis and osteogenesis, we constructed the shRNA sequences of SOX5, and shSOX5-1 with superior knock-down efficiency was selected for further experiments (Fig. 3A). Then, we transduced shSOX5 alone or combined with the EZH2 overexpression vector into the hFOB1.19 cells and HUVECs. Immunoblotting results displayed that shSOX5 resulted in reduced expression of SOX5 and EZH2, while further transduction with the EZH2 overexpression vector elevated the expression of EZH2 (Fig. 3B).

After osteogenic induction, the ALP and alizarin red S staining results showed that silencing of SOX5 increased ALP activity and calcium deposition, while additional overexpression of EZH2 reduced ALP activity and calcium deposition (Fig. 3D, E). Subsequent immunoblotting results displayed an increase in the expression of OCN, Runx2 and Collagen I in response to shSOX5 alone; however, these findings could be reversed by further transduction with EZH2 overexpression vector (Fig. 3C).

Transwell and capillary-like tube formation assays demonstrated an increased number of migrated cells and tube formation ability, accompanied by increased total network area, total length, and the number of branch points in hFOB1.19 cells transduced with shSOX5 alone, whose effects were abrogated by further EZH2 elevation (Fig. 3F, G and Supplementary Fig. 3A).

In summary, SOX5 inhibits HUVEC angiogenesis and hFOB1.19 cell osteogenesis by promoting the EZH2 expression.

### hucMSC-Exos augment HUVEC angiogenesis and hFOB1.19 cell osteogenesis by inhibiting EZH2

**Fig. 4:**
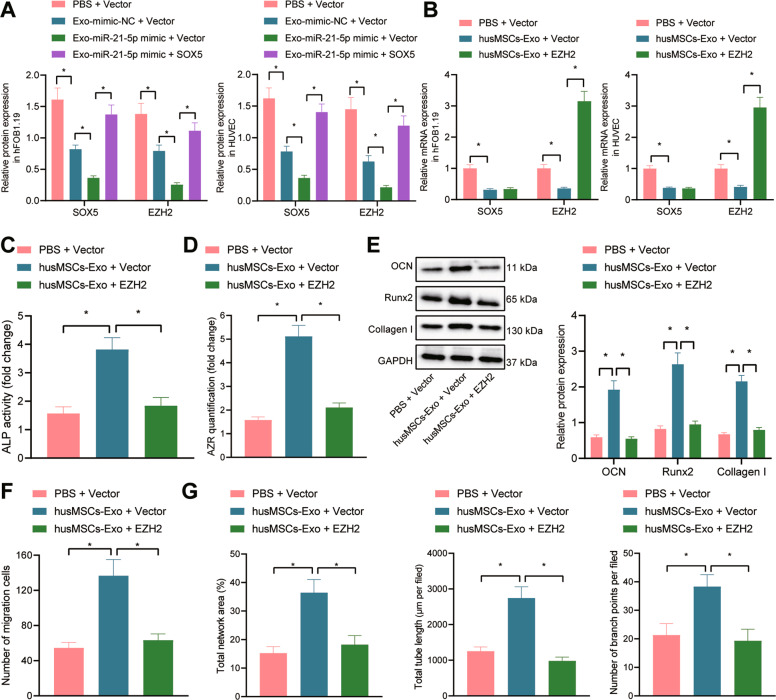
hucMSC-Exos enhance HUVEC angiogenesis and hFOB1.19 cell osteogenesis by inhibiting the expression of EZH2. **A** Immunoblotting analysis of SOX5 and EZH2 protein levels in hFOB1.19 cells and HUVECs; **B** SOX5 and EZH2 mRNA expression in hFOB1.19 cells and HUVECs determined with RT-qPCR; **C** quantitative analysis of ALP activity of hFOB1.19 cells; **D** quantitative analysis of calcium deposition of hFOB1.19 cells by alizarin red S staining; **E** Immunoblotting analysis of the expression of osteogenesis-related markers (OCN, Runx2 and Collagen I) in hFOB1.19 cells; **F** the migration of HUVECs determined with Transwell assay; **G** the tube-forming ability of HUVECs with total network area, total length, and the number of branch points determined with capillary-like tube formation assay. Cell experiment was repeated three times independently. *p < 0.05.

To investigate whether hucMSC-Exos containing miR-21-5p exerted function on angiogenesis and osteogenesis by regulating the SOX5/EZH2 axis, we co-cultured hucMSC-Exos with the hFOB1.19 cells and HUVECs. It was found that the expression of SOX5 and EZH2 was reduced in response to Exo-mimic-NC. Exo-miR-21-5p transduction notably diminished the expression of SOX5 and EZH2, while the effect was reversed by subsequent overexpression with SOX5 (Fig. 4A).

To clarify the role of hucMSC-Exos in angiogenesis and osteogenesis *via* the SOX5/EZH2 axis, we transduced EZH2 overexpression vector into the hFOB1.19 cells and HUVECs, respectively, followed by co-culture with hucMSC-Exos. As shown in Fig. 4B, hucMSC-Exo treatment reduced the levels of SOX5 and EZH2, while further transduction of EZH2 overexpression vector elevated the EZH2 expression, with no marked difference in the expression of SOX5. ALP and alizarin red S staining revealed increased ALP activity and calcium deposition after hucMSC-Exo treatment, while further overexpression of EZH2 abrogated the effects (Fig. 4C, D). As revealed by immunoblotting, a potent rise in OCN, Runx2 and Collagen I expression was induced in response to hucMSC-Exo, whose effects were negated by further overexpression of EZH2 (Fig. 4E). Transwell and capillary-like tube formation assays showed an increased number of migrated cells and enhanced tube formation ability, accompanied by increased total network area, total length, and the number of branch points after hucMSC-Exo treatment, while the effects were reversed by additional transduction of EZH2 overexpression vector (Fig. 4F, G and Supplementary Fig. 3B).

Therefore, hucMSC-Exos augment HUVEC angiogenesis and hFOB1.19 cell osteogenesis through inhibition of EZH2 expression.

### hucMSC-Exos containing miR-21-5p augment angiogenesis and osteogenesis in vivo

**Fig. 5:**
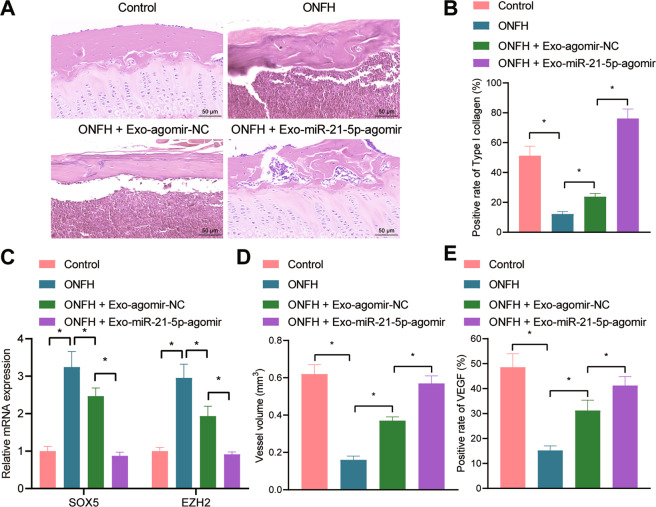
hucMSC-Exos augment angiogenesis and osteogenesis in vivo by delivering miR-21-5p. **A** H&E staining of femoral head tissues in rats; **B** immunohistochemistry of collagen I expression in femoral head tissues in rats; **C** SOX5 and EZH2 mRNA expression in rat femoral head tissues determined by RT-qPCR; **D** angiography of the vascular system in the femoral head tissues in rats by microfiber perfusion; **E** VEGF expression in the femoral head tissues in rats determined with immunohistochemistry. *n* = 6. *p < 0.05.

To define the role of hucMSC-Exos in vivo, a rat model of ONFH was established and treated with hucMSC-Exos. H&E staining of the femoral head tissues showed that compared with the sham-operated rats, the ONFH rats presented a higher number of empty cavities and coalescent bone cells in the trabecular bone cavity with sparse trabecular structure and a serious degree of necrosis. Exo-agomir-NC injection reduced the number of empty cavities or abscesses with poor necrotic trabecular bone structure in the ONFH rats, and further alleviated evidences were observed in the miR-21-5p-agomir-injected ONFH rats relative to Exo-agomir-NC (Fig. 5A).

Relative to sham-operated rats, the expression of collagen I was reduced in the ONFH rats, while its level was increased by treatment with Exo-agomir-NC. The Collagen I expression was profoundly increased in response to Exo-miR-21-5p-agomir relative to the Exo-agomir-NC (Fig. 5B). Bedsides, increased levels of SOX5 and EZH2 were witnessed in the ONFH rats relative to the sham-operated rats, while the effect was reversed by further administration of Exo-agomir-NC. Meanwhile, the Exo-miR-21-5p-agomir treatment induced marked reductions in the expression of SOX5 and EZH2 compared with the Exo-agomir-NC (Fig. 5C).

Subsequently, we evaluated the vascular system of the femoral head with angiography, which revealed a severely damaged vascular system of the ONFH rats compared with the sham-operated rats. Exo-agomir-NC treatment increased the volume and number of blood vessels in the ONFH rats, while the Exo-miR-21-5p-agomir injection potently increased the volume and number of blood vessels compared to the Exo-agomir-NC (Fig. 5D). Besides, immunohistochemical detection demonstrated a reduced expression of VEGF in the ONFH rats relative to sham-operated rats, while its expression was elevated after injection of Exo-agomir-NC. Meanwhile, the Exo-miR-21-5p-agomir resulted in an upregulated level of VEGF relative to Exo-agomir-NC (Fig. 5E).

Coherently, hucMSC-Exos containing miR-21-5p enhance angiogenesis and osteogenesis in vivo, thereby alleviating ONFH in rats.

## Discussion

The obtained findings provided evidence suggesting that hucMSC-Exos containing miR-21-5p stimulated HUVEC angiogenesis and hFOB1.19 cell osteogenesis by inhibiting the expression of EZH2, thereby alleviating ONFH.

Initially, our findings revealed that hucMSCs could augment HUVEC angiogenesis and hFOB1.19 cell osteogenesis with the secretion of Exos. Consistently, extracellular vesicles from MSCs increase the expression of vascular endothelial marker, VEGF to facilitate angiogenesis and bone regeneration in the context of bisphosphonate-related osteonecrosis of the jaw [21]. In vitro, human urine-derived stem cell-derived Exos could augment endothelial angiogenesis and inhibit apoptosis. In vivo findings from the same study revealed that the extracellular vesicles from USCs could radically alleviate angiogenesis impairment, reduce the apoptosis of trabecular bone and marrow cells, accompanied by improved bone microarchitecture of the femoral heads in rats under early exposure to glucocorticoids [22]. Our data also revealed that miR-21-5p was principally concentrated in hucMSC-Exos, its level would decrease in the cartilage tissues of ONFH patients, where the ability of Exos containing miR-21-5p to augment HUVEC angiogenesis and hFOB1.19 cell osteogenesis was evident. Moreover, existing research highlighted the enrichment of miR-21-5p in extracellular vesicles from MSCs in autoimmune diseases [23]. Additionally, hucMSC-Exos by delivery with miR-21 can potentially inhibit the osteocyte apoptosis in glucocorticoid-induced ONFH in rats [12]. Notably, elevated miR-21-5p can evidently improve osteogenic differentiation of progenitor cells [24]. Furthermore, miR-21-5p elevation has been identified to serve as a potential pro-osteogenesis regulator as it can improve ALP activity, enhance matrix mineralization and upregulate the levels of Runx2 and OCN to augment osteogenic differentiation and mineralization [25]. Similarly, our findings also demonstrated the ability of hucMSC-Exos containing miR-21-5p to increase ALP activity and calcium deposition, and the concentration of osteogenesis-related markers OCN, Runx2, and Collagen I.

Another finding of our study was that miR-21-5p targeted SOX5 and negatively regulated its expression, where the application of SOX5 silencing promoted HUVEC angiogenesis and hFOB1.19 cell osteogenesis. A similar regulatory relationship between miR-21-5p and SOX5 has been identified in melanogenesis [26]. Elevated expression of SOX5 has been evident in bone marrow-derived MSCs of postmenopausal osteoporosis patients and its overexpression inhibits the osteogenic differentiation of MSCs supported by reductions in ALP activity and osteoblast marker levels (Collagen I and Runx2) [27]. With the administration of EPO treatment, a correlation between the upregulation of chondrogenic marker SOX5 is apparent with increased angiogenesis and accelerated callus formation [28]. In subsequent analysis, our findings revealed the functionality of SOX5 to augment the transcription of EZH2. The positive targeting relationship between SOX5 and EZH2 has been previously identified in breast cancer [15]. As EZH2 expression is overexpressed in steroid-induced ONFH, inhibited EZH2 could suppress the apoptosis of osteocytes, which inhibits steroid-induced ONFH development [20]. EZH2 inhibitor could increase angiogenesis and subsequently exercise a stimulatory effect on osteogenic differentiation during bone healing [29]. Our evidence strongly supported the hypothesis that SOX5 suppresses HUVEC angiogenesis and hFOB1.19 cell osteogenesis by upregulating the EZH2 expression.

**Fig. 6:**
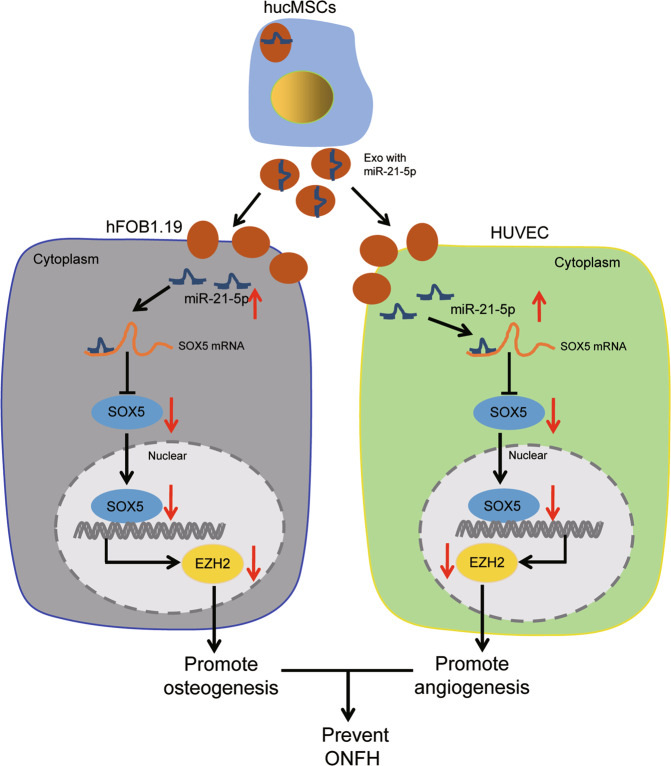
The mechanism graph of the regulatory network and function of hucMSC-Exos containing miR-21-5p on ONFH. hucMSC-Exos containing miR-21-5p augment HUVEC angiogenesis and hFOB1.19 cell osteogenesis by inhibiting the expression of SOX5/EZH2, thereby preventing ONFH.

Our work collectively supported that miR-21-5p, delivered by hucMSC-Exos, promoted HUVEC angiogenesis and hFOB1.19 cell osteogenesis by inhibiting the expression of SOX5/EZH2, thereby alleviating the ONFH (Fig. 6). Alternatively, researching miR-21-5p-containing hucMSC-Exos based on its regulatory molecules could potentially contribute to further understanding the mechanisms of ONFH and identifying novel therapeutic targets. Nevertheless, the specific downstream signaling pathways of the miR-21-5p remain to be determined and require further investigation in future experiments. Moreover, we only adopted a single dose of Exos in our experiments, thus the optimum dose and the times of injection necessitate further investigations.

## Materials and methods

### In silico prediction

The ONFH-related mRNA expression dataset GSE74089 was retrieved from the GEO database, and differential analysis was performed using the “limma” software package in the R language to screen out the differentially expressed mRNAs. Further, significantly upregulated genes in the GSE74089 dataset were intersected with the top 500 target genes by miR-21-5p using the starBase database, as well as the 100 genes predicted by TargetScan and miRDB databases. JASPAR CORE database was adopted to analyze the binding sites of SOX5 in the EZH2 promoter.

### Clinical sample collection

The tissue samples were harvested from 20 patients with ONFH and 20 patients with femoral neck fracture (normal controls) at The First Affiliated Hospital of Fujian Medical University. Those with osteoarthritis, rheumatoid arthritis, or other hip disorders diagnosed by measurement of clinical manifestations and radiologic imaging of the hip were excluded. All patients with ONFH and those with femoral neck fractures were diagnosed by at least two ONFH experts independently based on clinical manifestations and hip X-rays. All patients with femoral neck fractures underwent total hip replacement within 24 h of arrival. The cartilage tissues were isolated from the antero-superior portion of the femoral head, where the tissues had collapsed and the samples were preserved in liquid nitrogen for subsequent experiments.

### Cell culture and lentivirus transduction

HEK293T cells, human osteoblasts hFOB1.19, and HUVECs were acquired from the Procell Life Science & Technology Co., Ltd. (Wuhan, Hubei, China). These cells were cultured in DMEM containing 10% FBS, 100 U/mL penicillin, and 100 μg/mL streptomycin. miR-21-5p mimic, miR-21-5p inhibitor, mimic-NC, and inhibitor-NC were all purchased from GenePharma (Shanghai, China).

pHAGE-puro series plasmids and auxiliary plasmids pSPAX2, pMD2.G; pSuper-retro-puro series plasmids and auxiliary plasmids gag/pol and VSVG were co-transfected into the 293 T cells, followed by determination of the virus titer. The groups were as follows: Vector group (NC for transduction with lentiviral vector overexpressing SOX5), SOX5 group (transduction with lentiviral vector overexpressing SOX5), EZH2 group (transduction with lentiviral vector overexpressing EZH2), shNC group (transduction with lentiviral vector harboring NC of shRNA targeting SOX5), shSOX5-1 group (transduction with lentiviral vector harboring shRNA sequence-1 targeting SOX5), shSOX5-2 group (transduction with lentiviral vector harboring shRNA sequence-2 targeting SOX5). After 48 h of transduction, the GFP expression efficiency was observed under a fluorescence microscope.

A stably transduced cell line was successfully established. After 72-h virus transduction, the medium was replaced with a complete medium containing 2 μg/mL puromycin, and cells continued to be cultured for 5 days. The shRNA sequences were designed by Life Technologies and synthesized by GenePharma (Supplementary Table 1).

### Isolation, culture, and evaluation of hucMSCs

Human umbilical cords (*n* = 3) were isolated from healthy mothers after delivery and processed within 6 h of collection. Briefly, the umbilical cords were rinsed twice with PBS containing penicillin and streptomycin to remove any traces of blood. Next, 3–4 cm long sections of the umbilical cords were prepared in a petri dish, and cultured in DMEM/F-12 supplemented with 10% FBS, 1% GlutaMAX, 100 U/mL penicillin, and 100 μg/mL streptomycin. The sections were placed at 37 °C with 5% CO_2_ for 72 h and supplemented with fresh complete medium. Subsequently, half of the medium was renewed every 3 days, and the umbilical cord tissues were removed upon the appearance of the fibroblast-like cell set. The cells were trypsinized and passaged to attain 80–90% cell confluence. Early passage of hucMSCs (passage 2–6 times) was used in subsequent experimentation.

The morphology of the hucMSCs was observed under an inverted microscope (Leica DMI6000B, Weztlar, Germany). The differentiation potential of hucMSCs for osteogenesis, adipogenesis, and chondrogenesis was induced by the osteogenic, adipogenic, and chondrogenic mediums (Cyagen, Guangzhou, China). Alizarin red S staining was performed to examine the degree of calcium deposition on the 14th day of induction to assess the osteogenic differentiation of hucMSCs. Oil Red O staining was conducted to observe the concentration of lipid droplets on the 21st day of induction to evaluate the adipogenic differentiation of hucMSCs. Alcian blue staining was conducted to analyze the extracellular matrix on the 28th day of induction to assess the chondrogenic differentiation of hucMSCs.

The cell surface antigens of hucMSCs were determined using flow cytometry. Briefly, the hucMSCs were trypsinized and resuspended in 100 μL PBS. The cells were incubated with 1 μL of the fluorescein-conjugated monoclonal antibody PE-CD29 (555443, BD Biosciences, Sparks, MD), FITC-CD34 (555821, BD Biosciences), FITC-CD44 (555478, BD Biosciences), FITC-CD45 (561865, BD Biosciences), PE-CD73 (550257, BD Biosciences) and APC-CD90 (559869, BD Biosciences) for 1 h. The cells were then resuspended in 0.5 mL PBS, filtered using a nylon mesh, and subjected to a flow cytometric detection (BD Immunocytometry Systems) of the labeled isotype-matched antibodies (NC).

### Co-culture of hucMSCs and hFOB1.19 cells or HUVECs

hFOB1.19 cells or HUVECs (2 × 10^6^ cells/well) were plated in the basolateral chamber of the Transwell (3412, Corning, Midland, MI). Next, hucMSCs (4 × 10^5^ cells/well) or 10 μM GW4869 (an Exo production inhibitor) (D1692, Sigma, St Louis, MO) were plated in the apical chamber, followed by cell culture for 4–5 days. The fresh medium was renewed every 1–2 days, and the hFOB1.19 cells or HUVECs were collected for subsequent experimentation.

### Extraction and identification of Exos

hucMSCs were cultured overnight in the medium containing 10% Exo-free serum. Upon attaining 80–90% cell confluence, the supernatant was collected. The cells were centrifuged at 2000 × *g* for 20 min at 4 °C to eliminate cell debris, and the collected supernatant was also centrifuged at 100,000 × *g* for 1 h at 4 °C. Next, the pellet was suspended in serum-free DMEM containing 25 mM HEPES (pH = 7.4), and subjected to high-speed centrifugation. The supernatant was removed, and the pellet was preserved at −80 °C for subsequent use.

The morphology of hucMSC-Exos was observed under a TEM. Briefly, 30 μL of Exos was added to a copper mesh. The copper mesh was added with 30 μL of phosphotungstic acid solution (pH 6.8), counter-stained at room temperature for 5 min, dried using an incandescent lamp, and finally photographed under a TEM.

The expression of the Exo surface markers in hucMSC-Exos was identified by immunoblotting. Briefly, the Exo particles were dissolved in RIPA buffer and quantitatively measured using the bicinchoninic acid (BCA) kit (Thermo Fisher Scientific, Rockford, IL). The antibodies used were: TSG101 (ab125011 (Abcam, Cambridge, UK), CD63 (ab134045, Abcam), CD81 (ab109201, Abcam) and Calnexin (ab22595, Abcam).

Dynamic light scattering was performed to assess the diameter of the Exos using the Zetasizer Nano-ZS90 instrument (Malvern, Worcestershire, UK) with an excitation wavelength of 532 nm.

### Cy3 fluorescently labeled hucMSCs co-cultured with hFOB1.19 cells or HUVECs

Cy3-labeled miR-21-5p (miR-21-5p-Cy3) was purchased from GenePharma and transfected into the hucMSCs based on instructions of Lipofectamine 2000 reagent (11668019, Invitrogen, Carlsbad, CA) to evaluate the delivery of miR-21-5p into Exos. Next, hucMSCs expressing Cy3-miR-21-5p were co-cultured with hFOB1.19 cells or HUVECs in a Transwell chamber (3412, Corning) for 2–4 days. The hFOB1.19 cells or HUVECs were fixed with 4% paraformaldehyde, and permeabilized with PBS containing 0.5% Triton X-100, after which the nuclei were stained with DAPI (C1002, Beyotime, Shanghai, China). Finally, the hFOB1.19 cells and HUVECs were observed under a confocal microscope.

### Exo uptake evaluation

The extracted hucMSC-Exos were labeled in strict accordance with instructions of the PHK67 labeling kit (KH67GL, Sigma). The hFOB1.19 cells and HUVECs were subjected to overnight culture, co-cultured with 10 μg PHK67 labeled Exos for 24 h, and then immersed in 4% paraformaldehyde. The cells were permeabilized with 2% Triton X-100 and blocked with 2% BSA. After staining with DAPI (2 μg/mL), fluorescence expression was observed under a FV-1000/ES confocal microscope.

### RT-qPCR

The total RNA content was extracted from the tissues using Trizol (16096020, Invitrogen). The cDNA of the miRNA with PolyA tail was synthesized using the PolyA tailing detection kit (B532451, Sangon, Shanghai, China) containing the universal PCR primer R and U6 universal PCR primer R. The cDNA of mRNA was reversely-transcribed using a reverse transcription kit (RR047A, Takara, Japan). The samples were subjected to RT-qPCR reaction in a real-time fluorescent quantitative PCR instrument (ABI 7500, ABI, Foster City, CA). RT-qPCR was performed according to provided protocols by the TaqMan Gene Expression Assay (Applied Biosystems, Foster City, CA). The miRNA in the Exos was normalized to syn-cel-miR-39-3p (Supplementary Table 2). Relative expression was quantified by the 2^−ΔΔCt^ method.

### Immunoblotting

The total protein content was extracted using the RIPA lysis buffer (Beyotime) with protease inhibitors, followed by protein concentration quantitation using a BCA kit (20201ES76, Yeason, Shanghai, China). Protein was separated by PAGE and then transferred onto PVDF membrane by a wet transfer. Following blocking with 5% BSA, membranes were probed with the primary antibodies against SOX5 (ab94396, Abcam, 1:1000), EZH2 (ab186006, Abcam, 1:2000), osteocalcin (OCN; ab133612, Abcam, 1:1000), Runx2 (ab236639, Abcam, 1:1000), Collagen I (ab34710, Abcam, 1:2000), GAPDH (ab8245, Abcam, 1:3000; internal reference) overnight at 4 °C. The membrane was then re-probed with diluted secondary antibodies against HRP-labeled goat anti-rabbit IgG (ab6721, Abcam) or goat anti-mouse IgG (ab6789, Abcam) for 1 h at room temperature. Following visualization in chemiluminescence reagent, protein quantitative analysis was conducted by the ImageJ software.

### Alizarin red S and ALP staining

After attain 60–70% cell confluence, the medium was replaced with an osteogenic differentiation medium (HUXMA-90021, Cyagen) for continuous culture. On the 21st day after incubation, the cells were fixed using 4% paraformaldehyde, and stained with Alizarin Red (G8550, Solarbio, Beijing, China) to determine the degree of osteogenic differentiation. On the 7th day of incubation, the ALP activity was evaluated with an ALP color development kit (CBA-300, Cell Biolabs). The images were acquired under an optical microscope (IX 70, Olympus, Tokyo, Japan).

### ChIP assay

ChIP assay was conducted using the EZ-Magna ChIP TMA kit (Millipore, Billerica, MA) as described previously [30]. HEK293T cells were cross-linked with 1% formaldehyde. Cells were supplemented with the protease inhibitor and ultrasonicated to obtain 200–1000 bp chromatin fragments. DNA fragments were added into 900 μL of ChIP Dilution Buffer and 20 μL of 50× PIC. After centrifugation, the supernatant was collected as Input. DNA fragments were immunoprecipitated with 1 μL of rabbit anti-SOX5 (ab94396, Abcam), while the NC group was supplemented with 1 μL of rabbit anti-IgG (ab172730, Abcam). Next, 20 μL of 5 M NaCl was added for de-crosslinking to recover the DNA content, from which the enriched chromatin fragments were detected by fluorescence real-time PCR (F: 5′-GCTAGTTATTAAATTCAT-3′, R: 5′-GTCATGTAAACTGAGAT-3′).

### Dual-luciferase reporter experiment

A dual-luciferase reporter gene plasmid containing the SOX5 3’UTR sequence (full-length wild-type WT, mutant MUT) and EZH2 promoter sequence (WT, MUT with the binding site ATTTTTA mutated to ACGCGGC) was constructed, respectively. The dual-luciferase reporter plasmids were co-transfected with the corresponding plasmids into the 293T cells. After 48 h of transfection, the luciferase activity was detected using a Dual-Luciferase^®^ Reporter Assay System (E1910, Promega, Madison, WI). The firefly luciferase activity was normalized to renilla luciferase activity.

### Capillary-like tube formation assay

The pre-cooled and melted Matrigel (Corning) was spread evenly to the bottom of the pre-cooled 24-well plate, transferred to the cell culture incubator, and allowed to rest 30 min to facilitate the solidifying process of Matrigel. Next, 2.5 × 10^4^ HUVECs were seeded onto a 24-well plate, supplemented with PBS or hucMSC-Exos to attain a final concentration of 50 μg/mL [3] for cell culture for 8 h, after which the plate observations were documented under an optical microscope (Olympus IX 73).

### Transwell migration assay

HUVECs were trypsinized using a 24-well plate Transwell chamber (8 μm, Corning), resuspended in serum-free DMEM (Gibco), with a density adjusted to 3 × 10^5^ cells/mL. Three chambers were set for each group. Each chamber was supplemented with 200 μL of cell suspension, while the basolateral chamber was supplemented with 700 μL of 10% DMEM. After 48 h incubation at 37 °C with 5% CO_2_, cells were fixed with methanol and stained with 0.05% crystal violet (G1062, Solarbio). The migrated cells were counted and photographed under an optical microscope.

### Development of a rat model of ONFH

Healthy male Sprague-Dawley rats (weighing 250-300 g; aged 20-24 weeks old) were provided by the Shanghai Experimental Animal Center of the Chinese Academy of Sciences (Shanghai, China) and housed individually in separate cages in a SPF animal laboratory. Rats had ad libitum access to food and water with a relative humidity of 60–65% and temperature of 22–25 °C under a 12-h light and dark cycle. The rats were acclimated for 1 week before the experiment. The health of the rats was observed before the experiment.

Within 60 min prior to the operation, all rats were injected intramuscularly with 4 mg/kg gentamicin, and then injected intraperitoneally with 1 mL/kg of 3% pentobarbital sodium for anesthesia (n = 6). After skin shaving and local disinfection, a longitudinal incision was made over the greater trochanter. The gluteus maximus was detached from the bone in direction of its bundles. The anterolateral joint capsule was transected along the trochanteric ridge, and the ligamentum teres was incised to isolate the femoral head. The periosteum at the base of the femoral neck was incised with the reflected fibers of the joint capsule at 1 mm intervals using a number 11 blade. For the surgery group, a ligature (Vicryl#1; Ethicon, Somerville, NJ) was wrapped securely around the left femoral neck to disrupt the vascular supply. For the sham-operated group, the frenulum was not knotted, and thus ischemia was not induced. After the displacement of the femoral head, the joint capsule and gluteal muscles were sutured with Vicril#2-0, and the skin was sequentially sealed with nylon 2-0. Two weeks after induction of ischemia, 100 μg (dissolved in 200 μL PBS) of Exos (Exo-agomir-NC and Exo-miR-21-5p-agomir) or the same amount of PBS were injected daily *via* the tail vein. After eight weeks, the rats were euthanized by CO_2_ asphyxiation.

### Immunohistochemistry

The fixed decalcified femoral head was embedded in paraffin and sliced using an ultramicrotome. The sections were subsequently deparaffinized with xylene, rehydrated using graded alcohol, and incubated with 3% hydrogen peroxide to terminate any endogenous peroxidase activity. After antigen retrieval in sodium citrate, sections were blocked using 10% normal goat serum. The sections were subjected to overnight incubation with antibodies against Collagen I (ab34710, Abcam) or VEGF (ab1316, Abcam) at 4 °C. The following day, the sections were incubated with the secondary antibody for 1 h at room temperature, after which the immunoreactivity was detected using the DAB kit (Invitrogen).

### Angiography

After euthanasia and fixation of rats on the operating table, the abdominal aorta was exposed and perfused with heparin saline and formalin for 20 min under appropriate pressure. Next, with rupture of the capillary, the rats were gradually injected with micro-fil (Flow Tech, Carver, MA) and preserved overnight in a refrigerator at 4 °C after perfusion. Next, the femur was isolated, fixed with formalin, and decalcified for 1 month. Finally, the samples were scanned using the SkyScan-1176 microcomputer tomography (μCT) system (Bruker Micro-CT), and the vascular system of the femoral head was reconstructed and quantified.

### H&E staining

H&E staining was performed using the H&E staining kit (C0105, Beyotime). Briefly, the sections were stained with hematoxylin for 5–10 min, and then counter-stained with eosin for 30 s to 2 min. The sections were observed under an inverted microscope.

### Statistical analysis

All experimental data were analyzed using the SPSS 19.0 statistical software (IBM Corp., Armonk, NY), with *p* < 0.05 as a level of statistical significance. Measurement data were summarized as mean ± standard deviation. Differences between two groups were compared using an unpaired *t* test, while those among multiple groups were analyzed using a one-way analysis of variance, followed by Tukey’s post hoc test.

## Supplementary information

Original Data File

Supplementary materials (supplementary figures and tables)

## Data Availability

Data sharing is not applicable to this article as no datasets were generated or analyzed during the current study.
