# Supplementary figures and images for: Pro-angiognetic and pro-osteogenic effects of human umbilical cord mesenchymal stem cell-derived exosomal miR-21-5p in osteonecrosis of the femoral head
Source: Cell Death Discov. 2022 Apr 25;8:226. doi: 10.1038/s41420-022-00971-0 (PMC9039080; doi:10.1038/s41420-022-00971-0)

fig3C


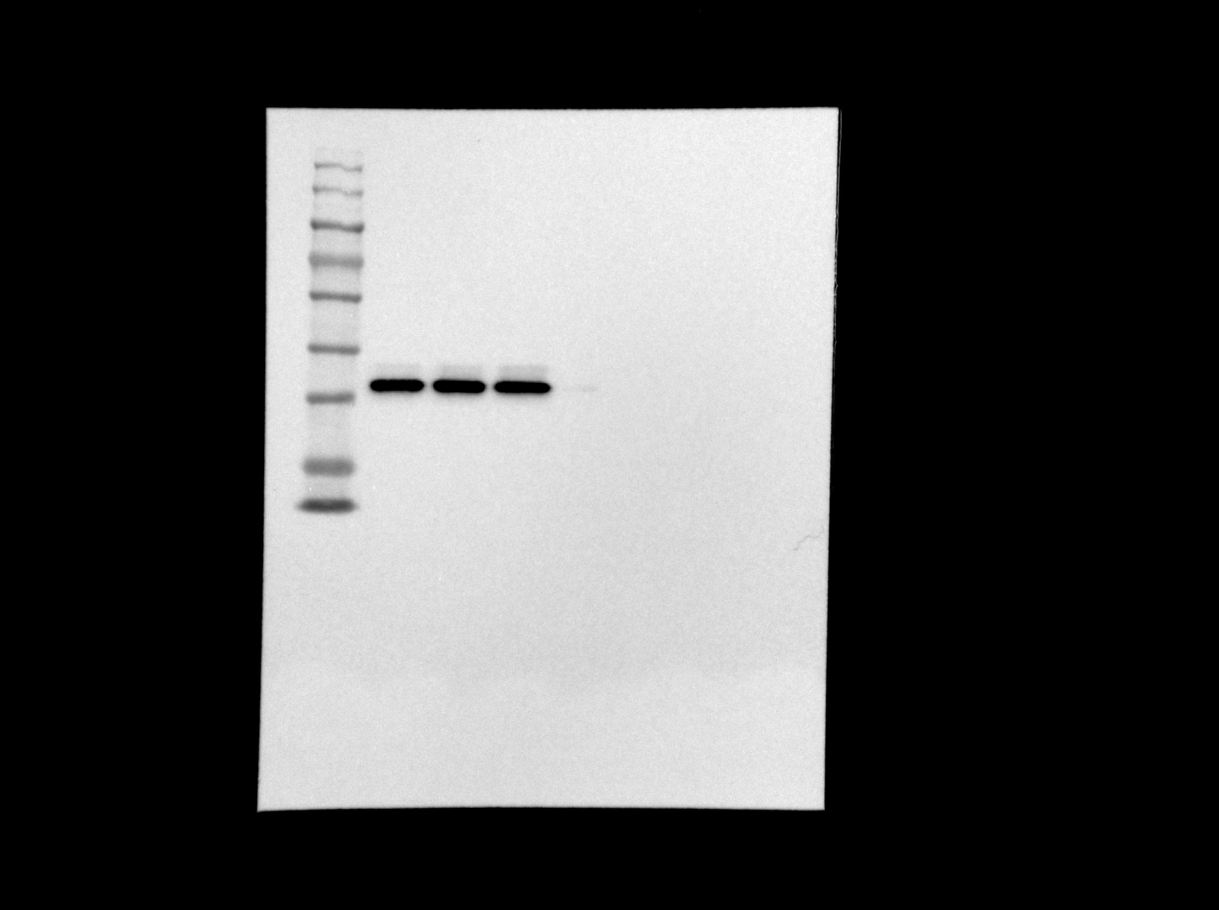

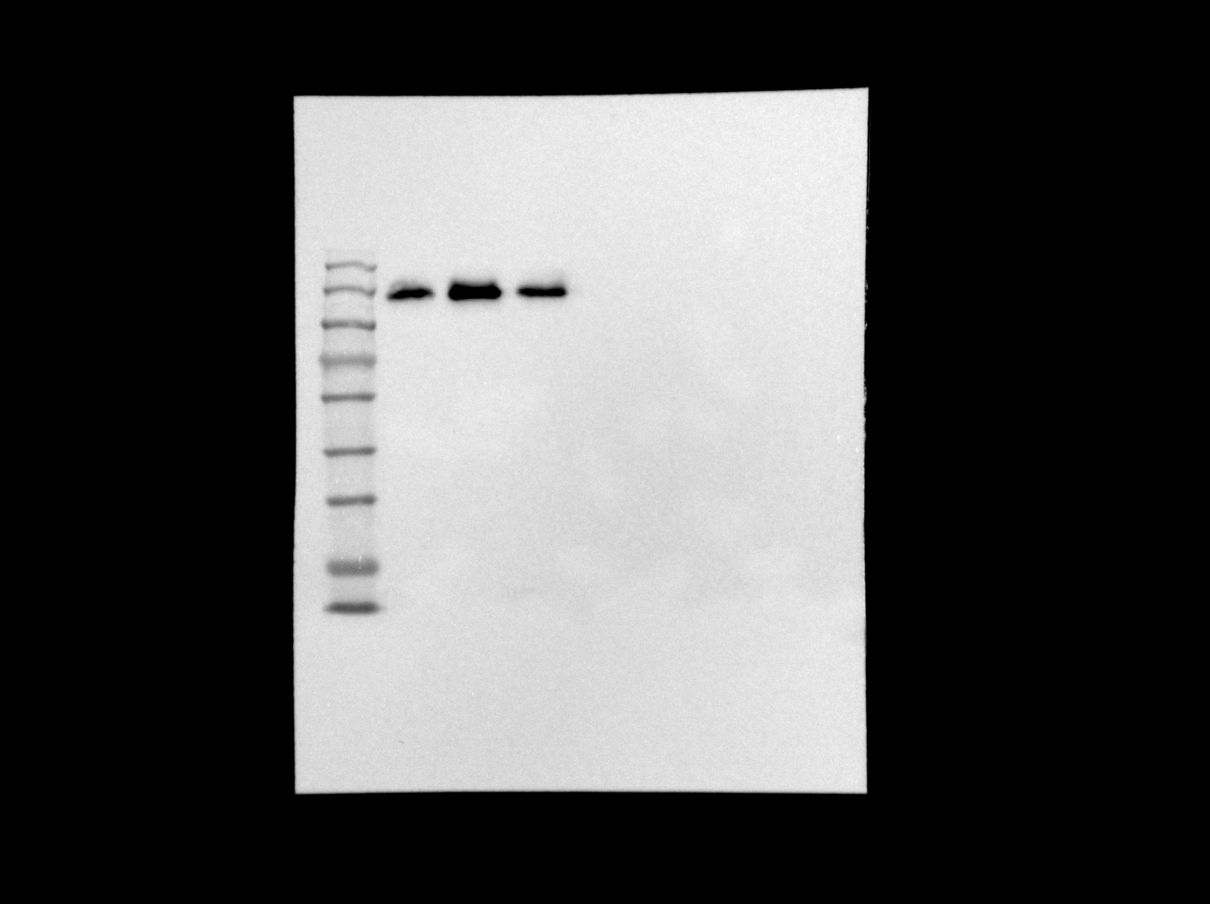

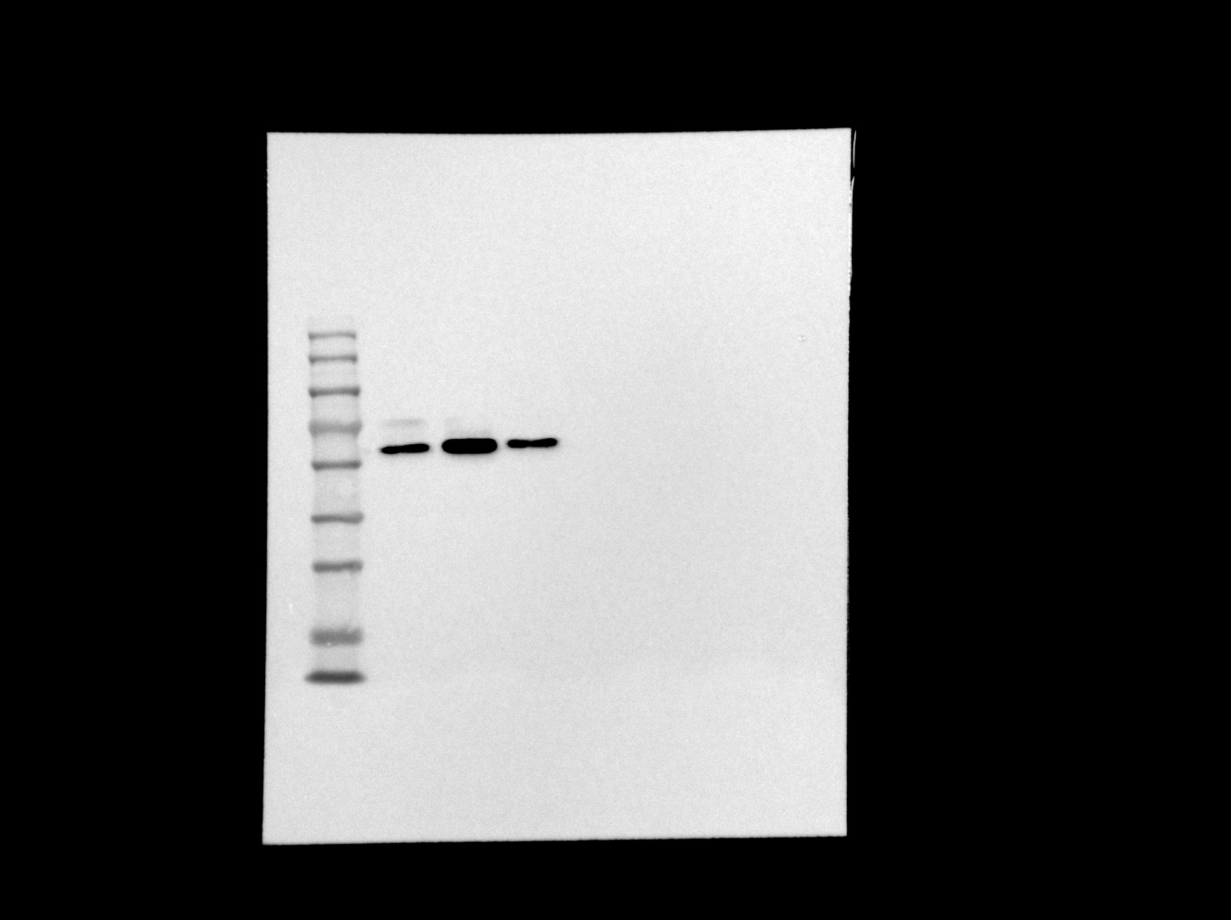

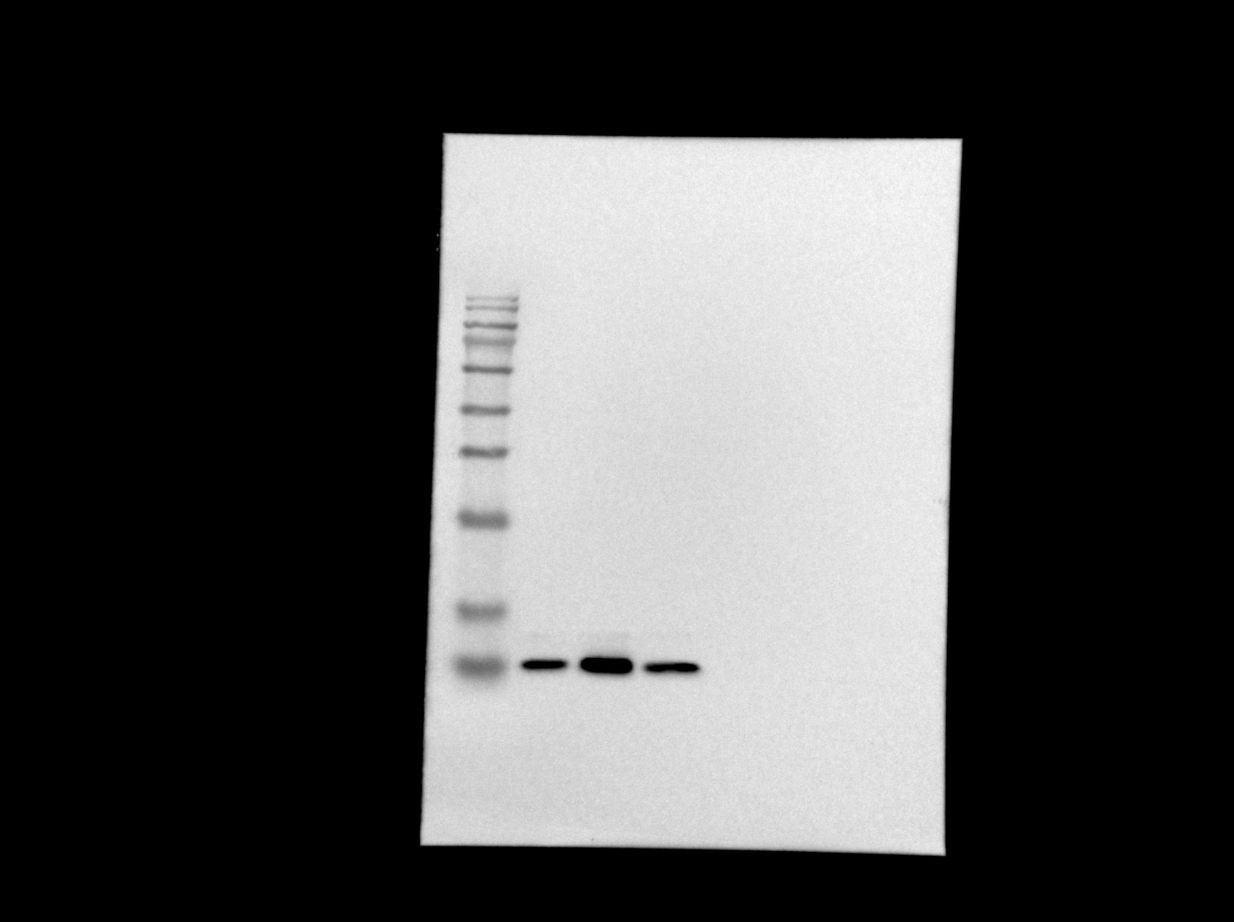


fig4E


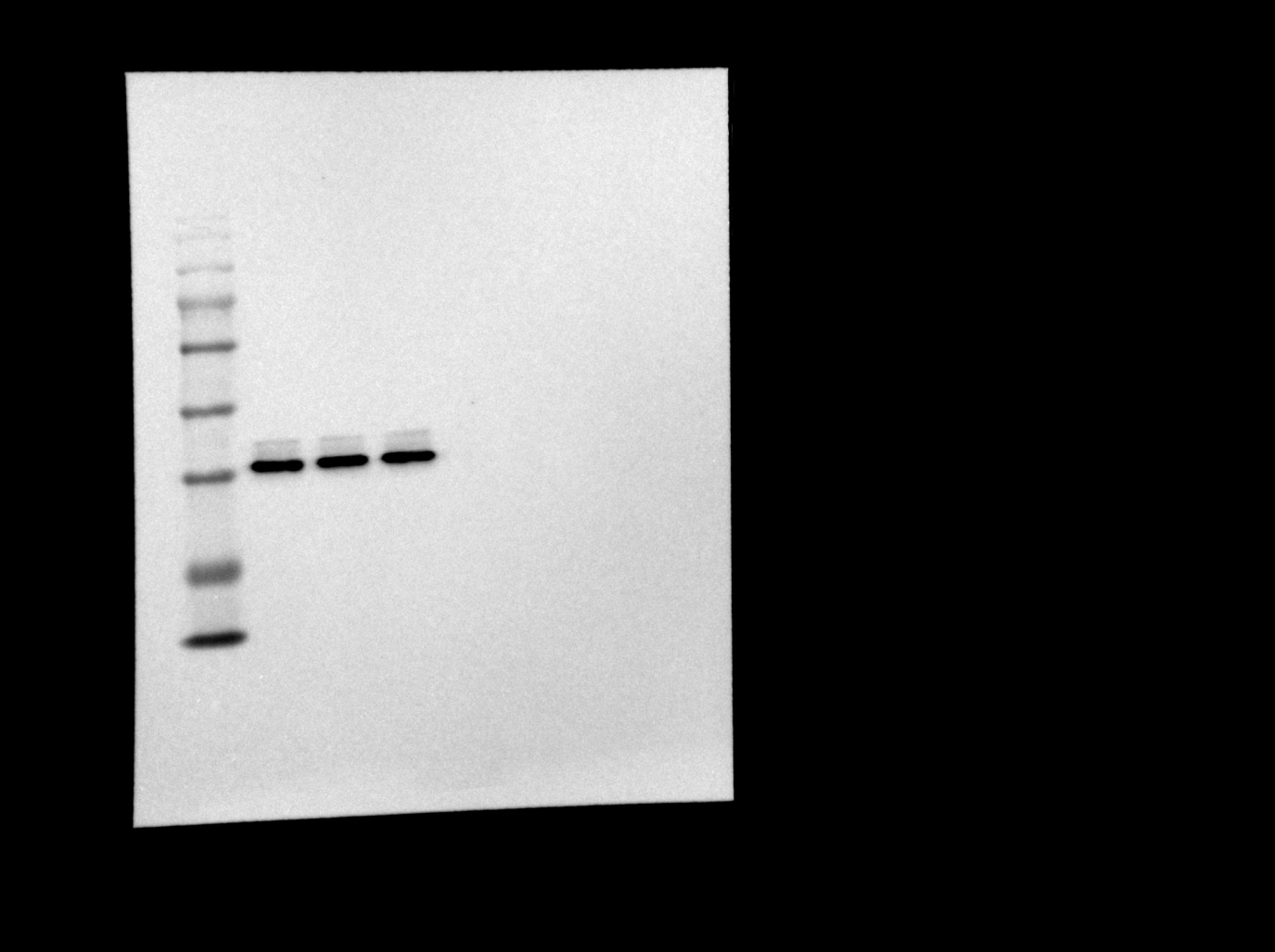

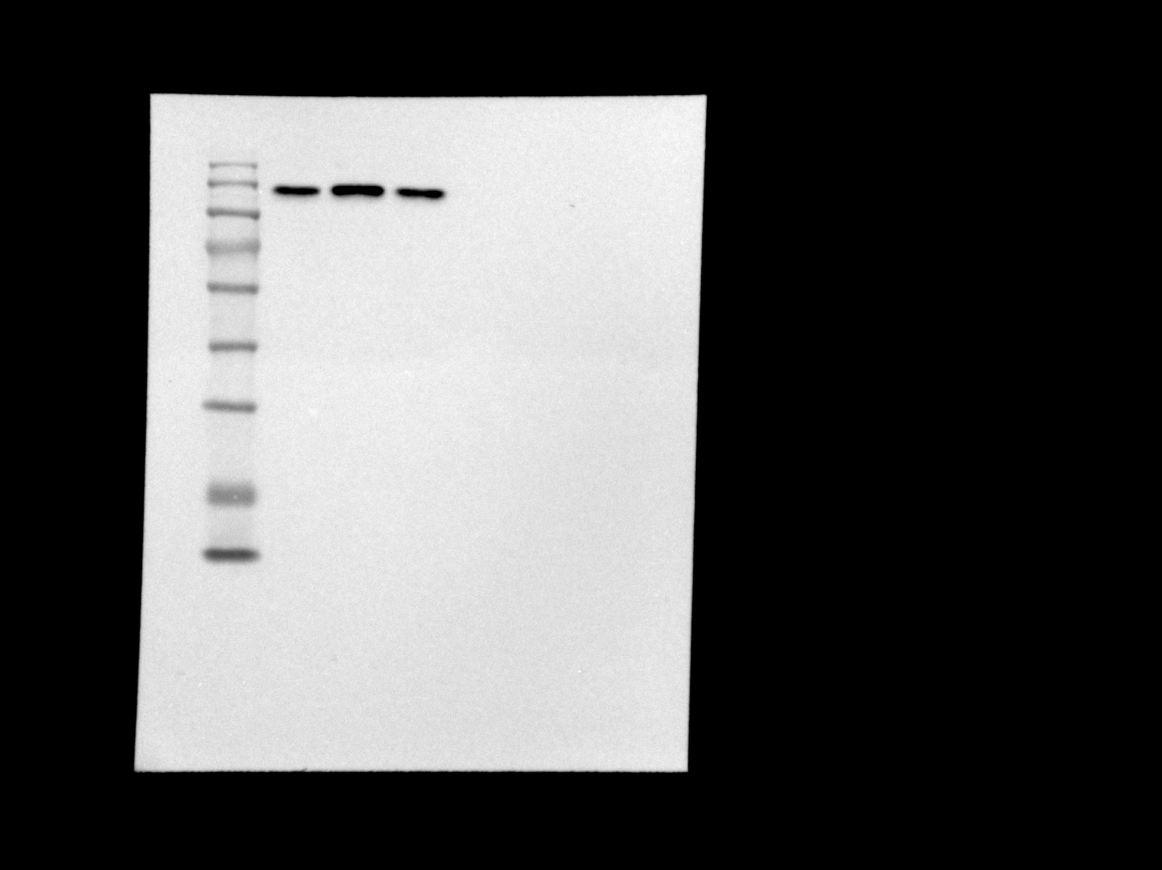

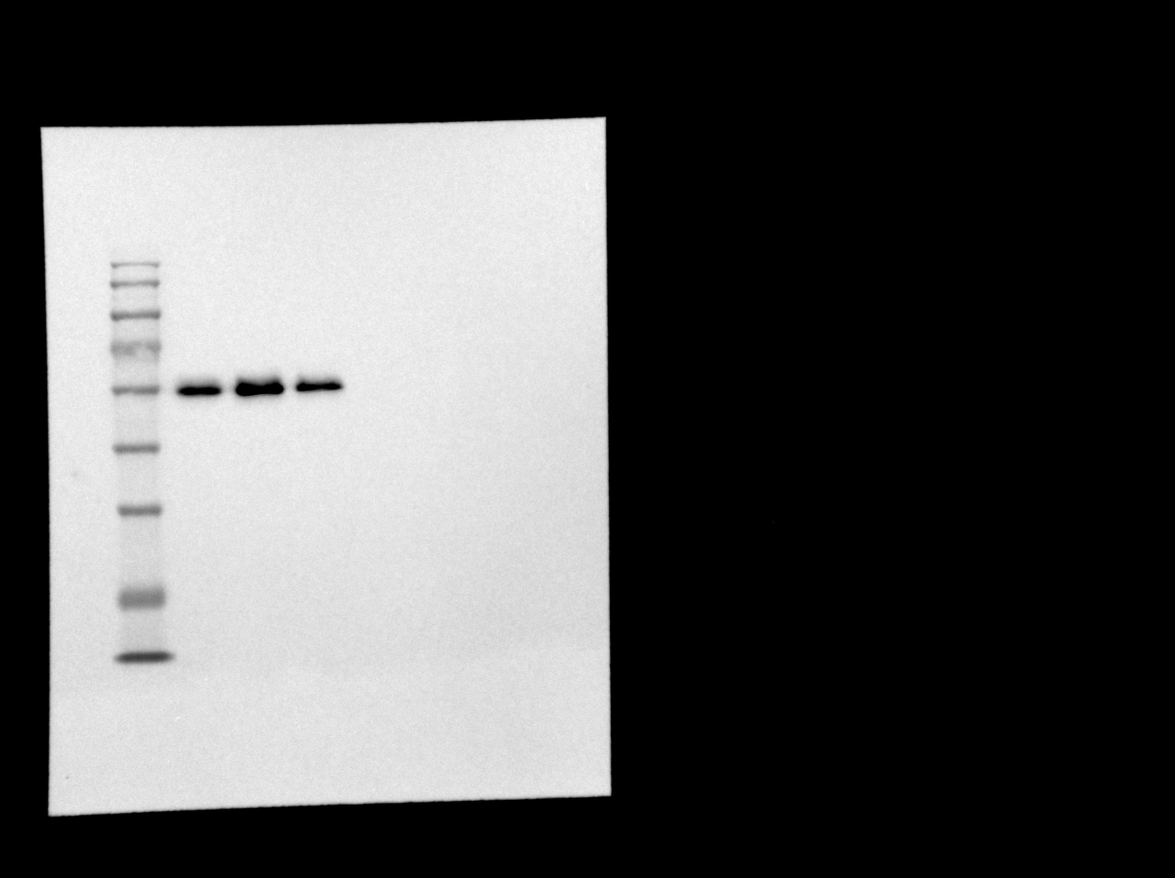

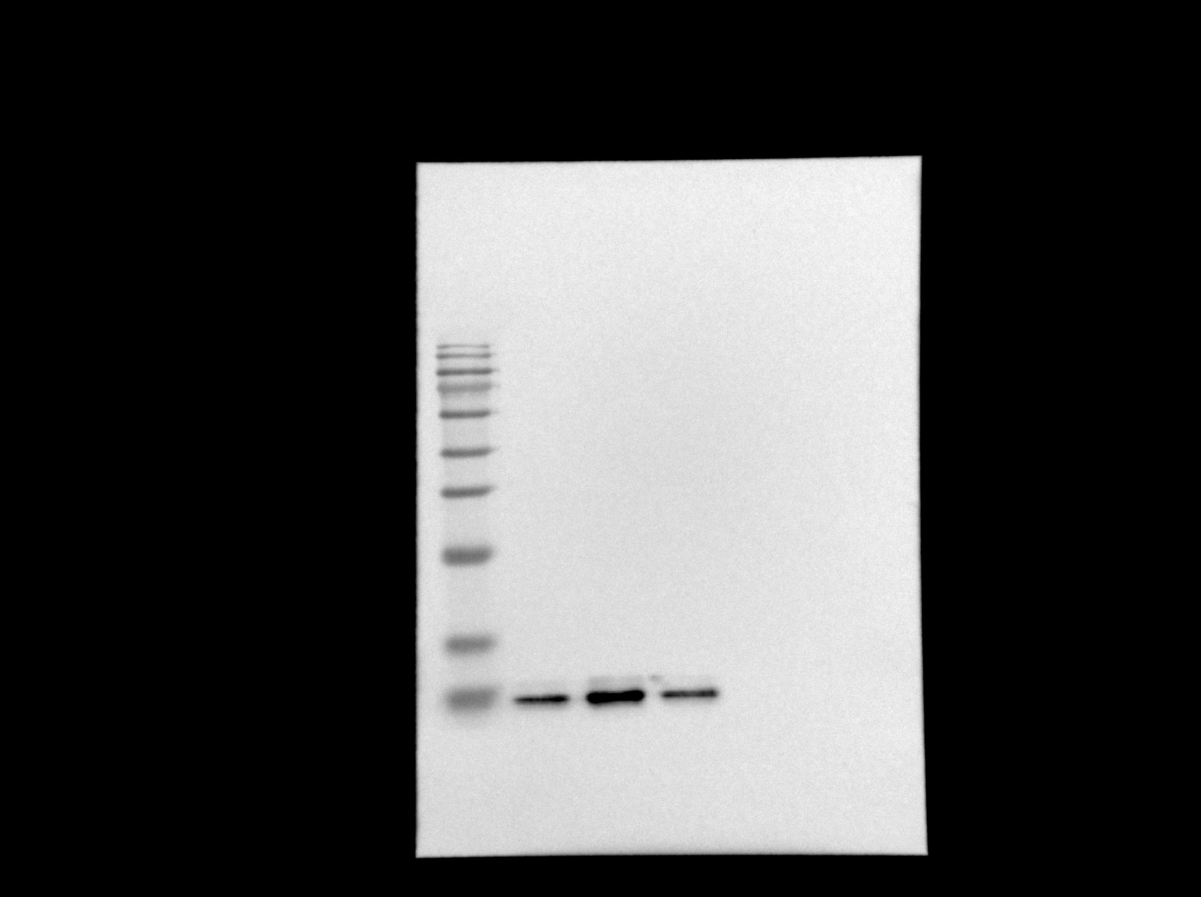


figS1F


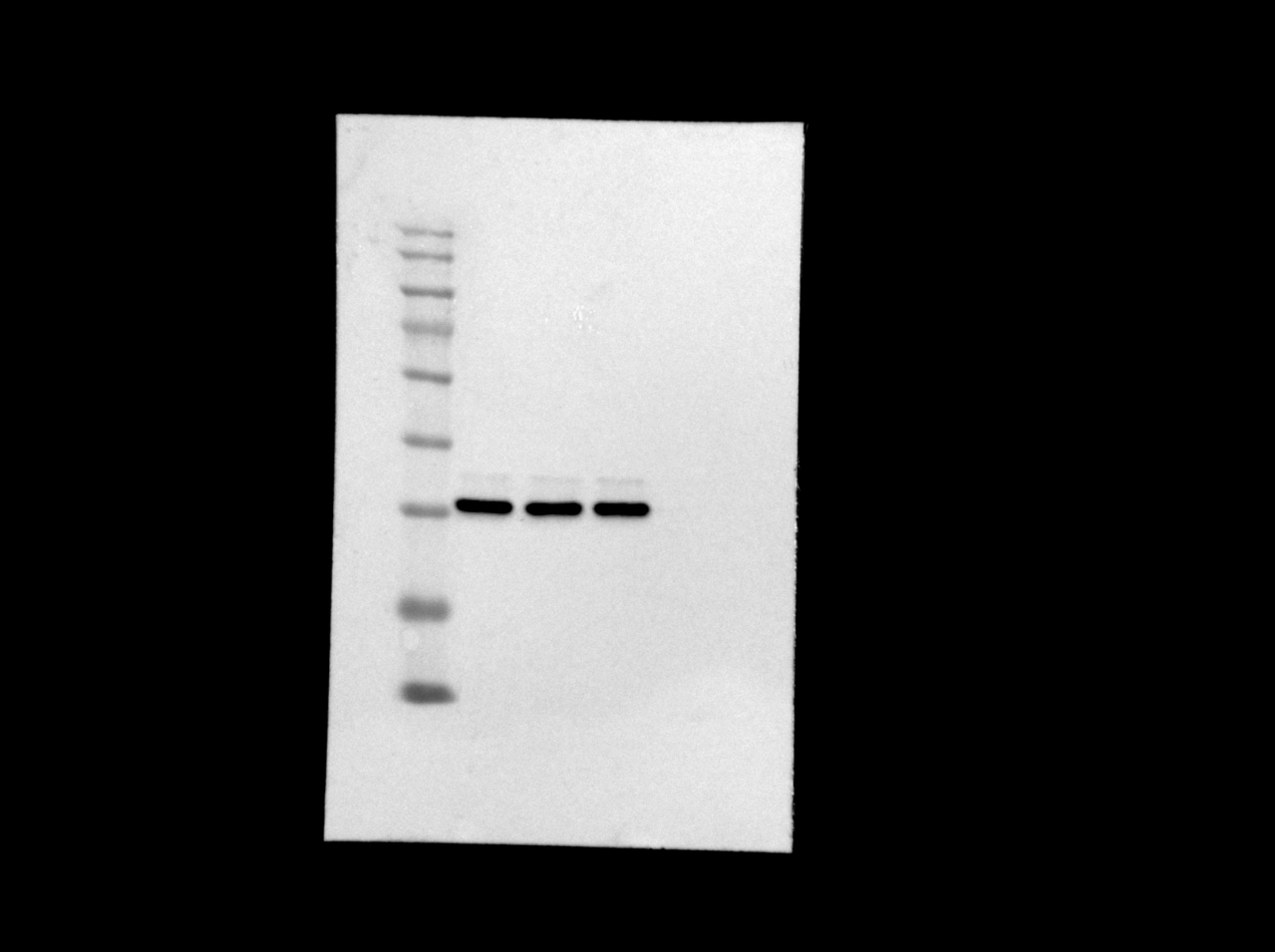

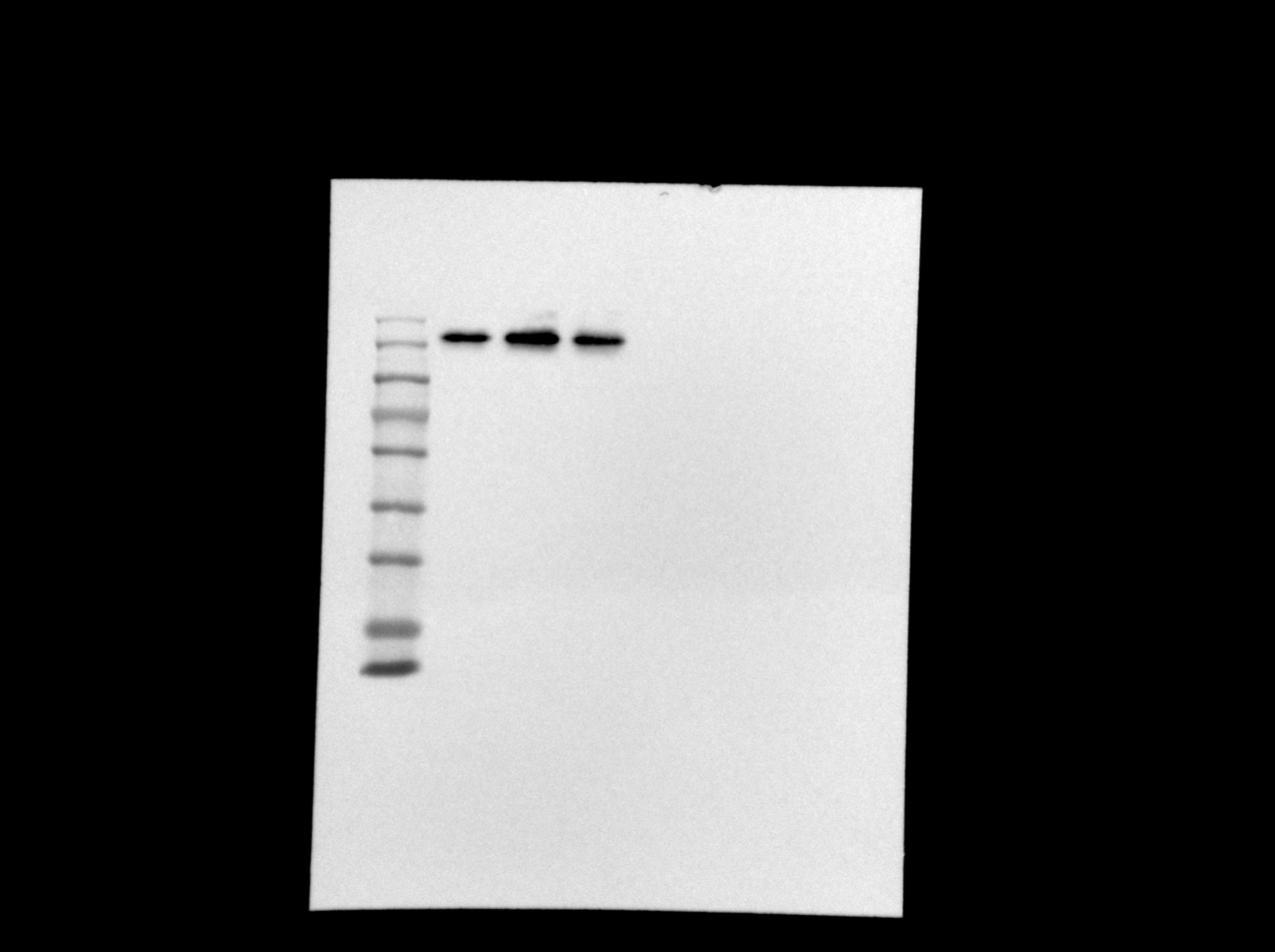

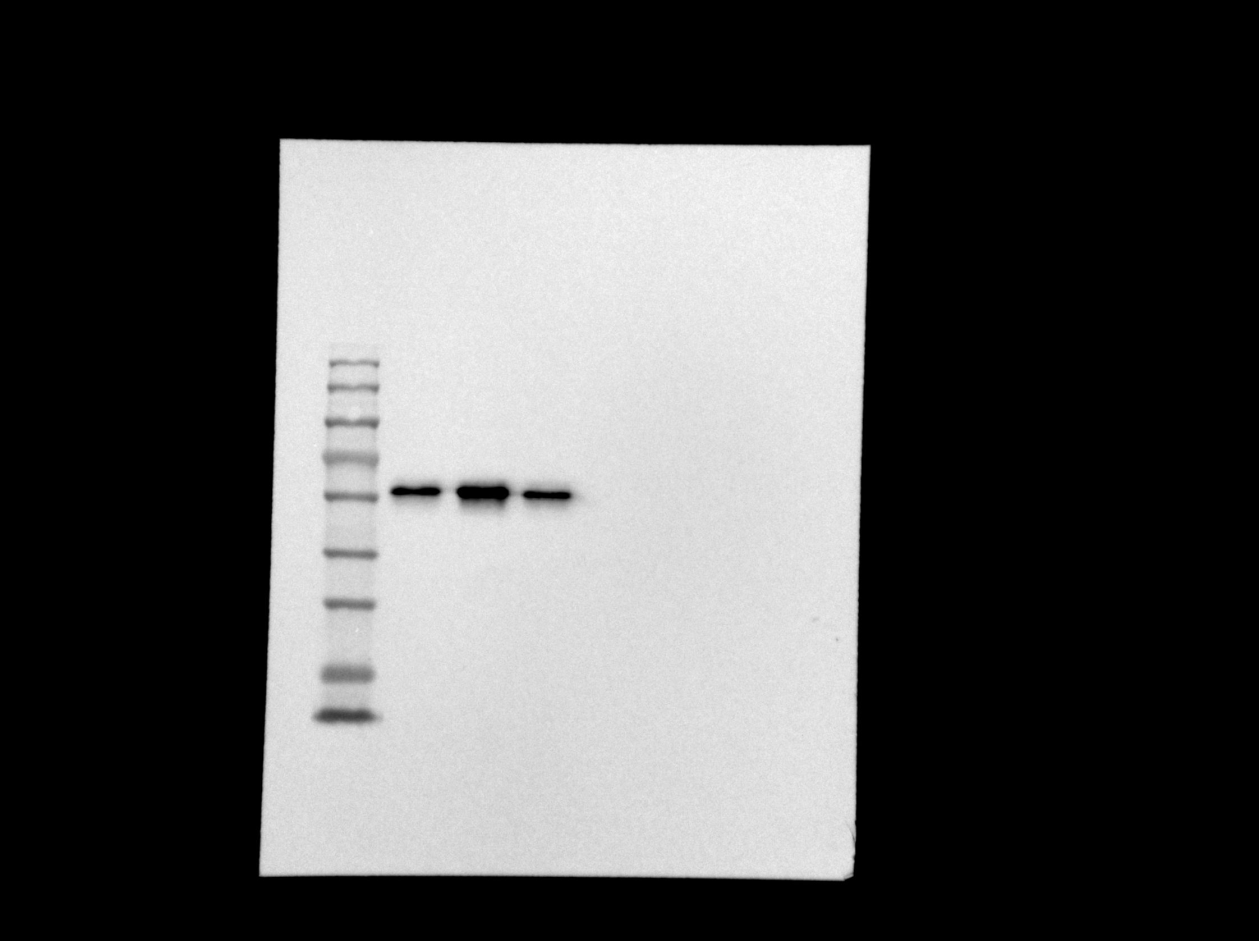

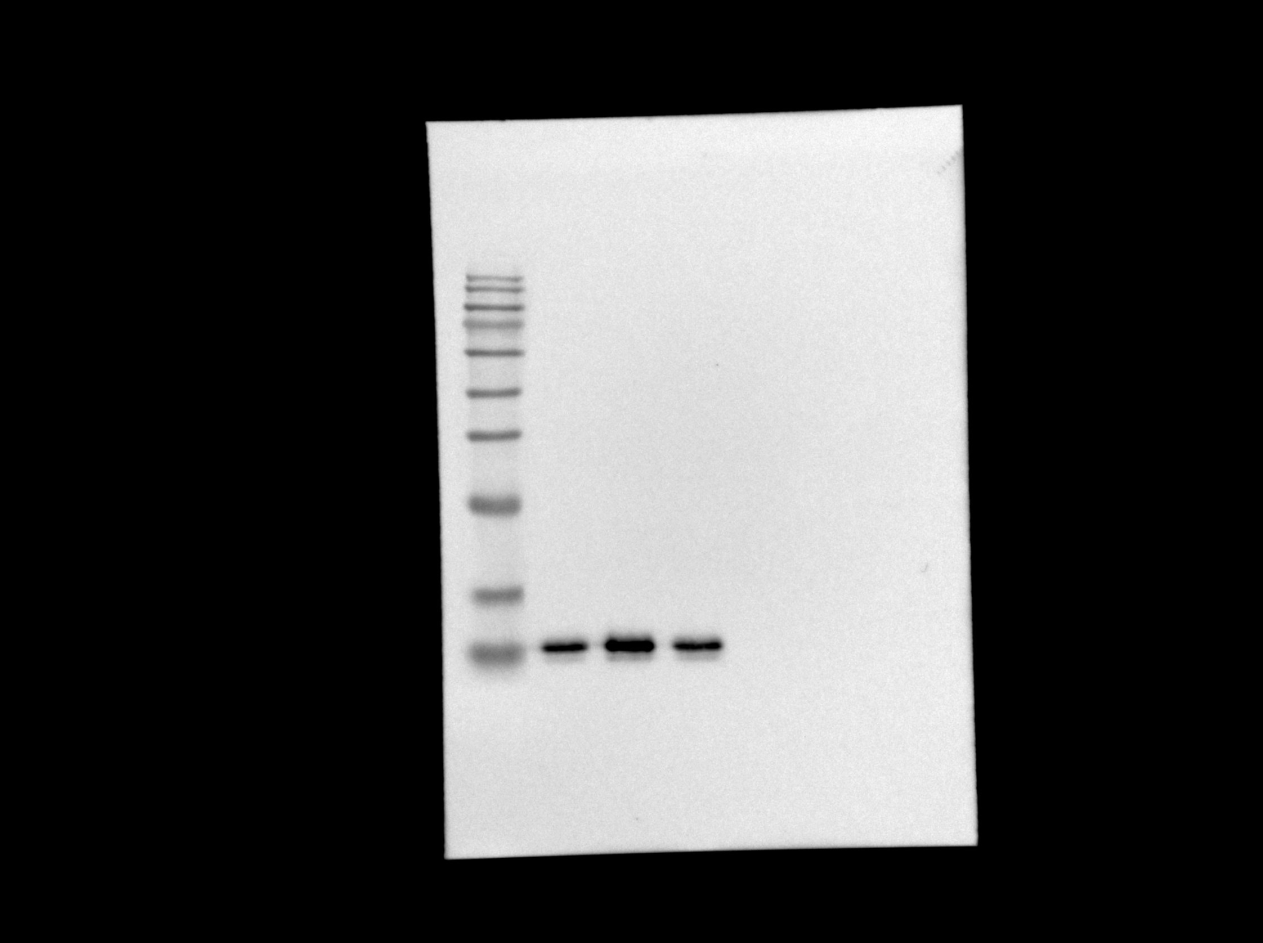


figS2D


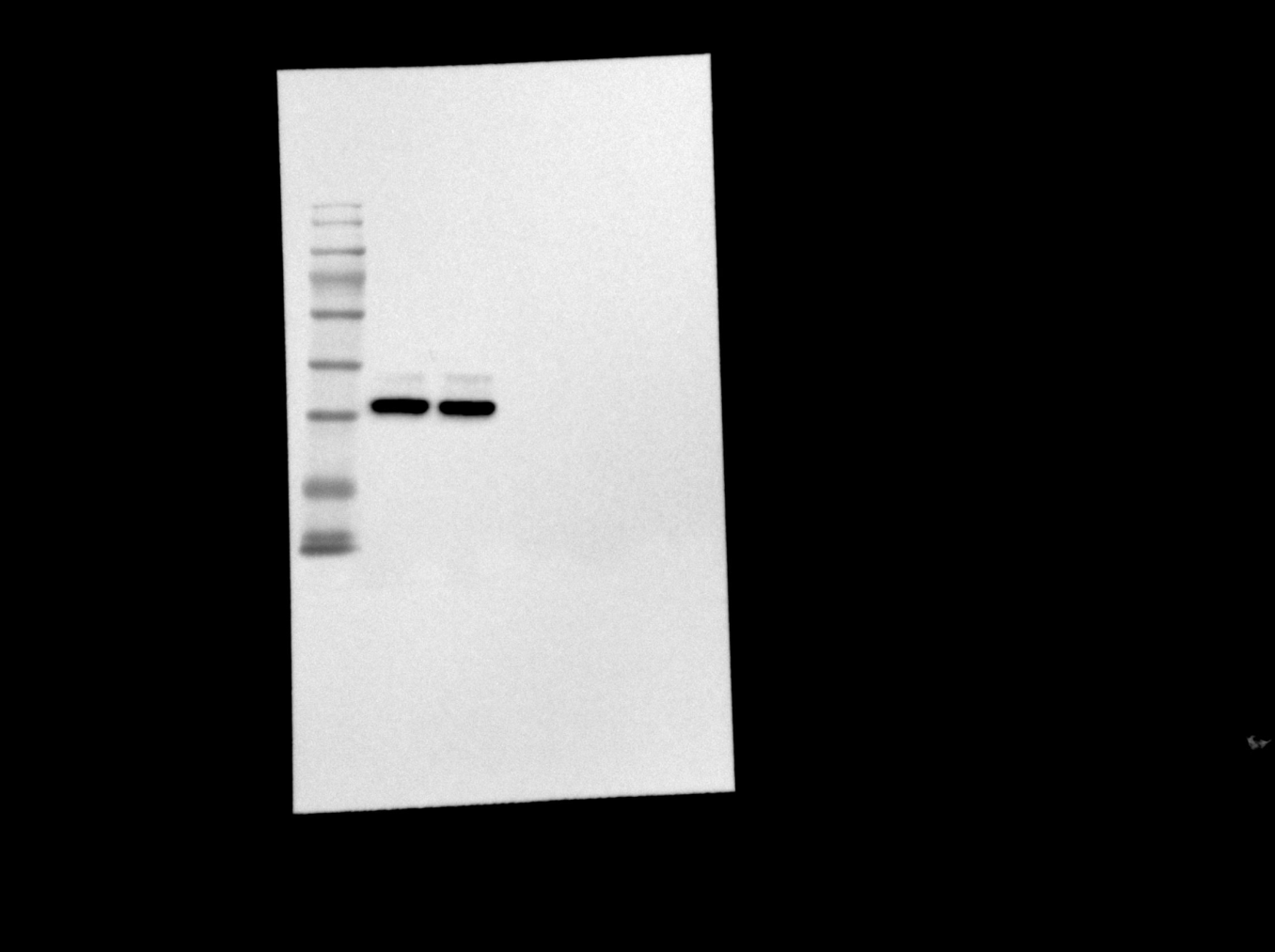

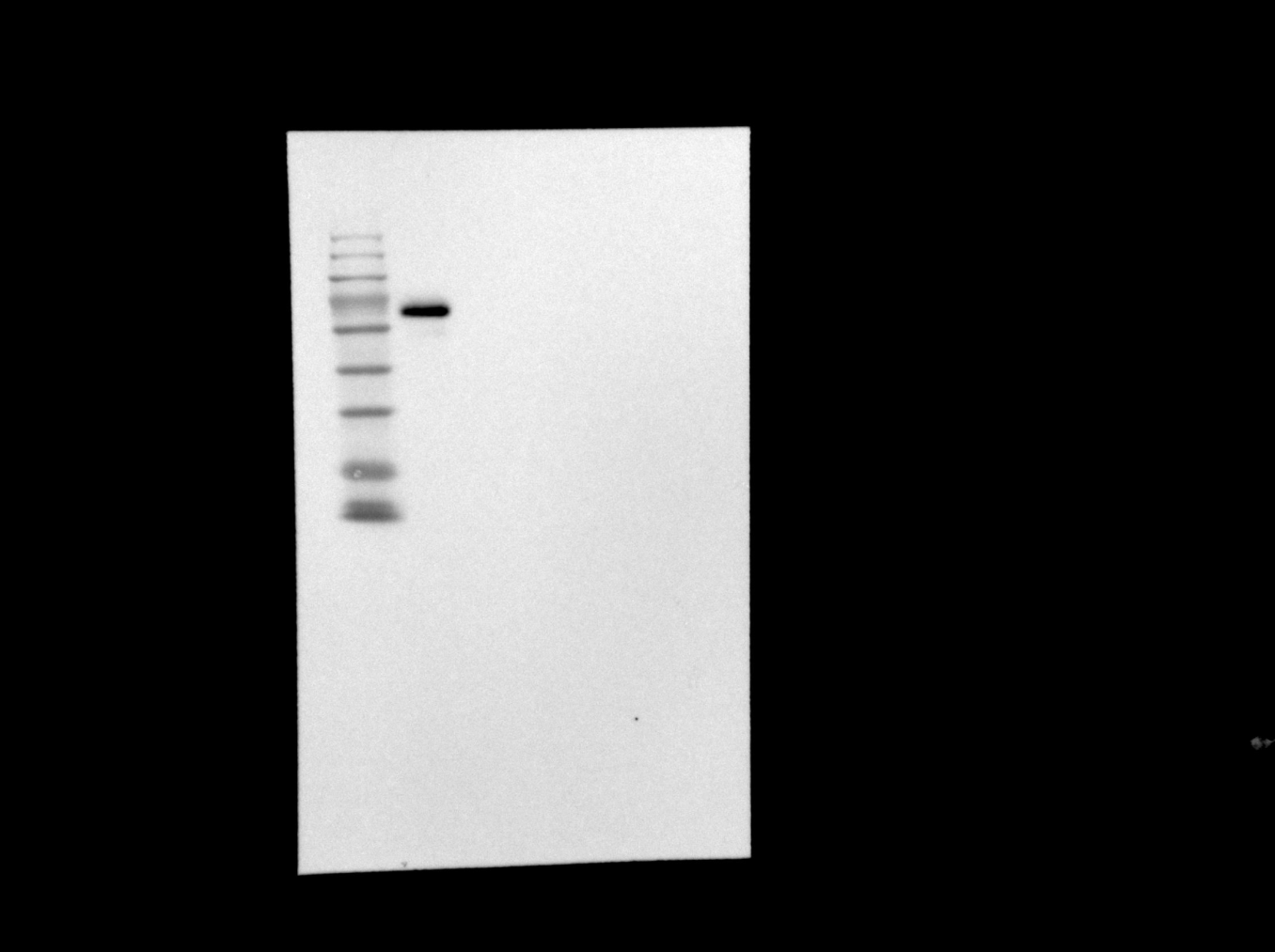

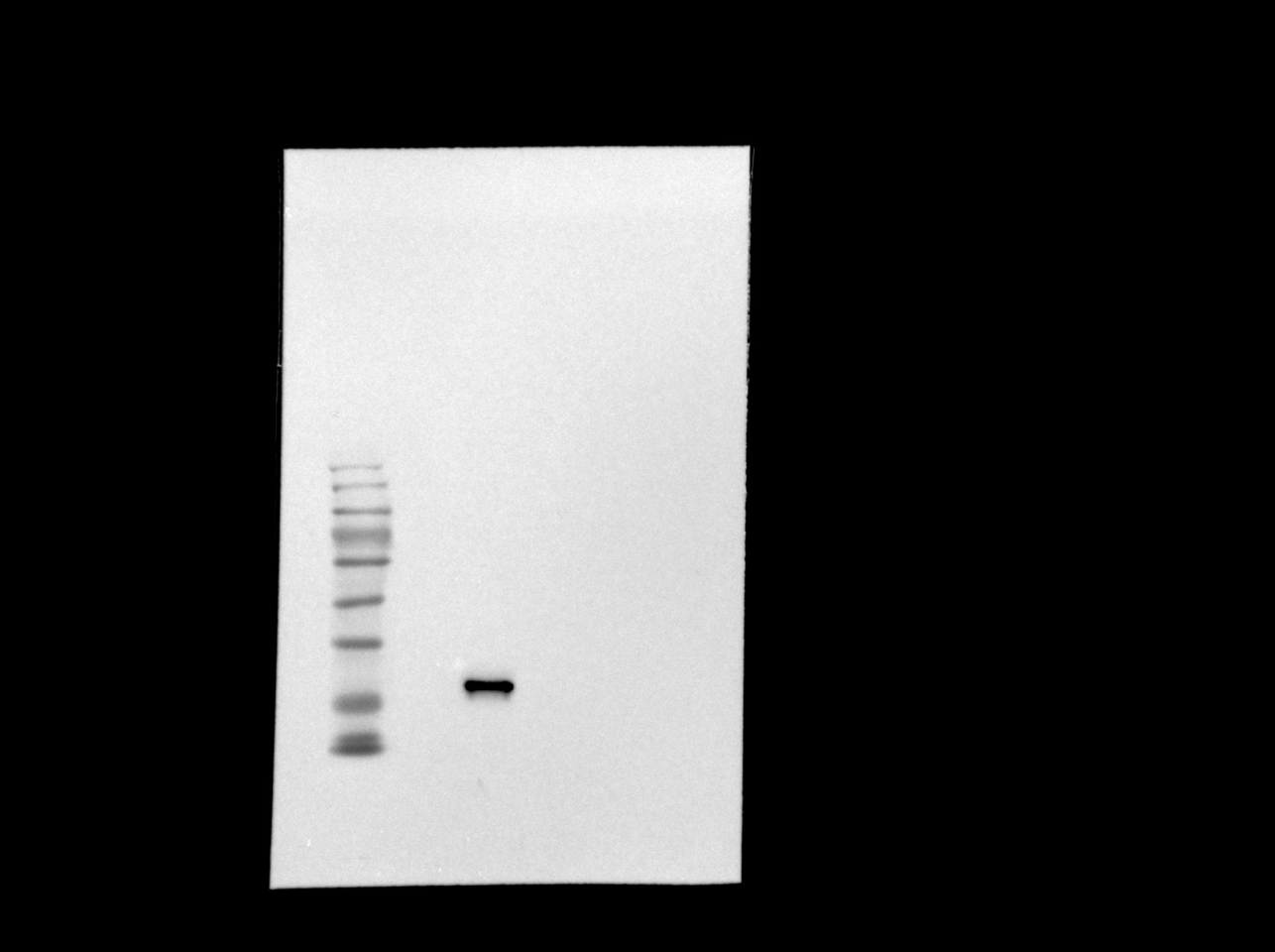

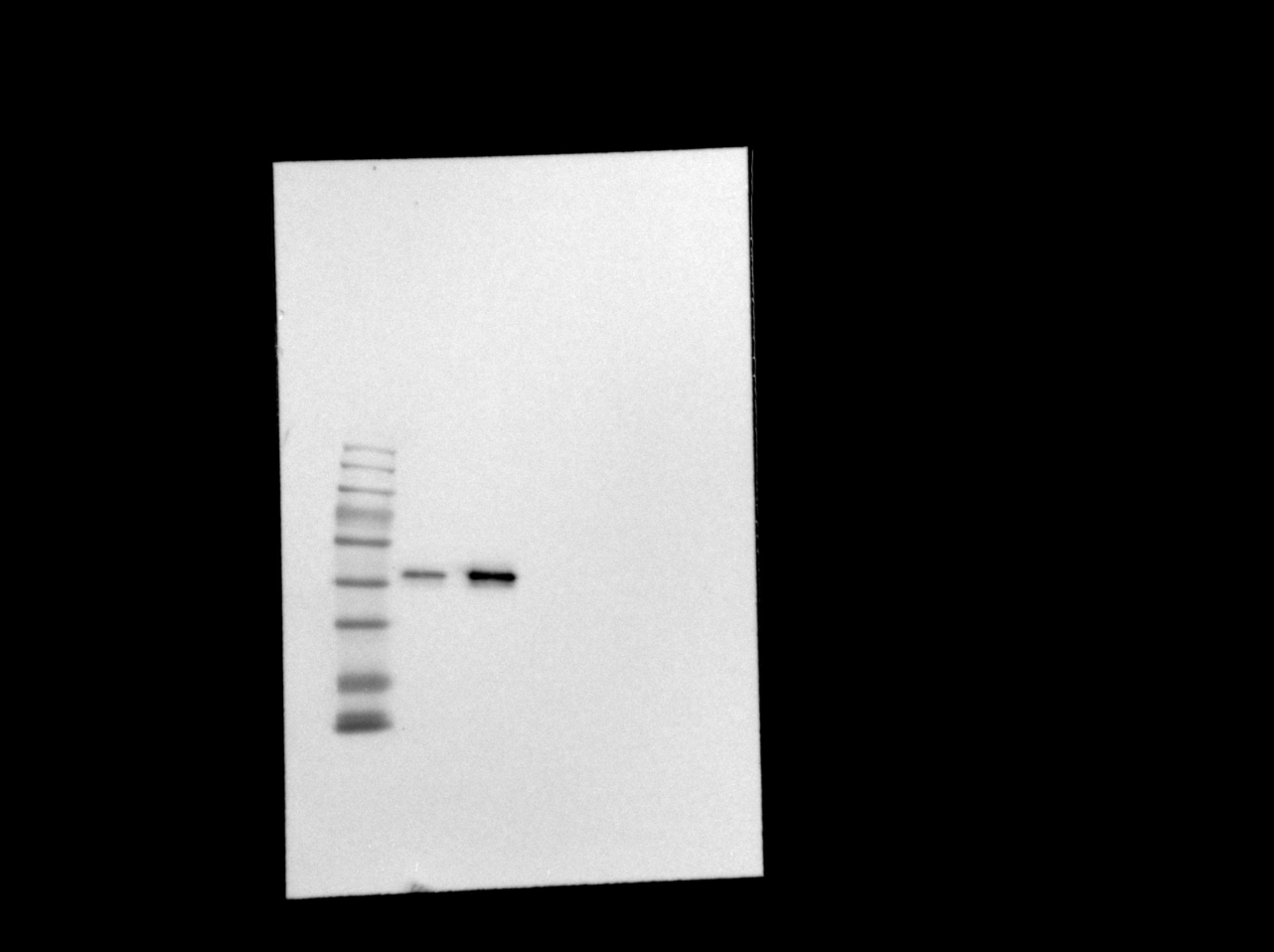

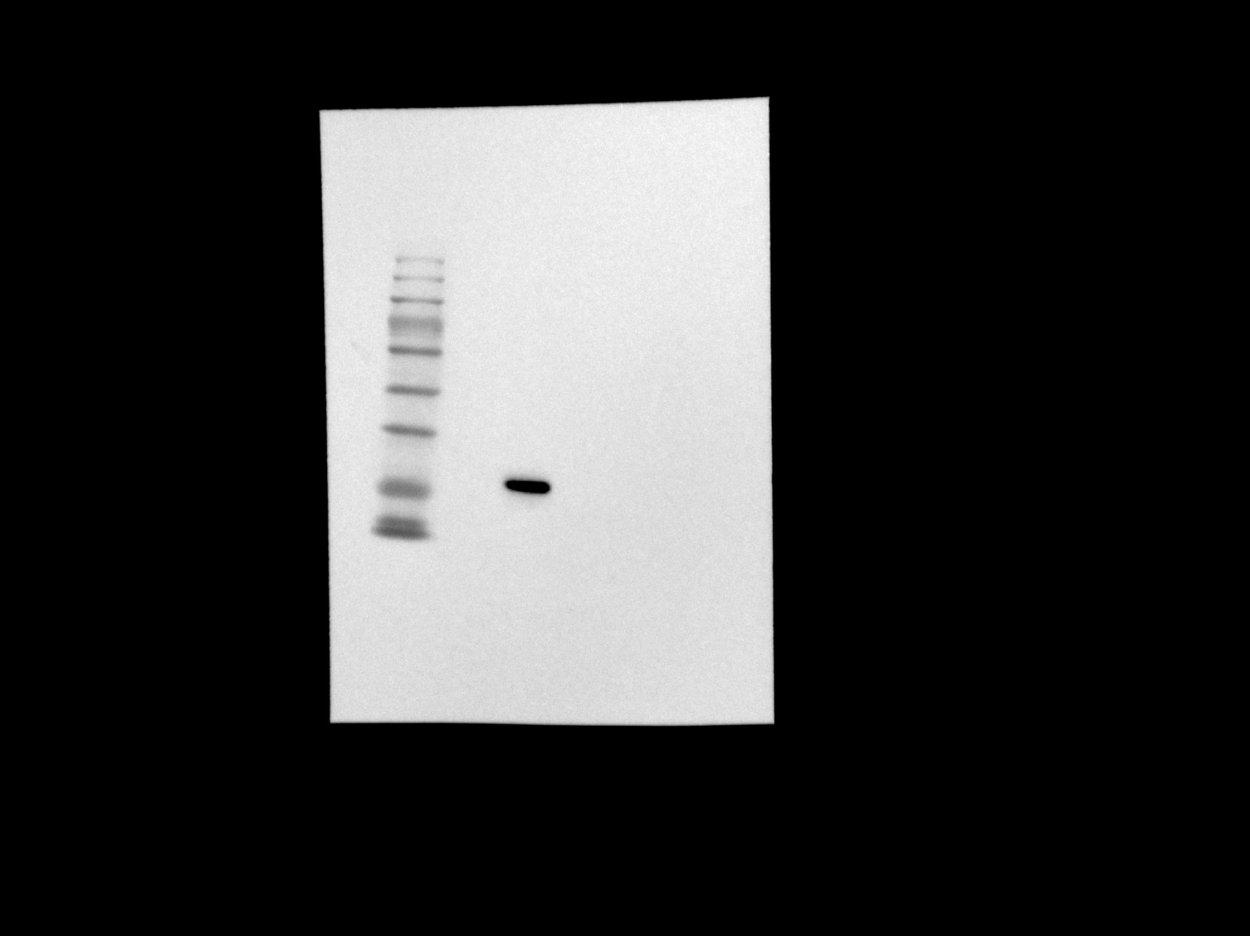

Supplement: Supplementary file 1 — Original Data File [file 41420_2022_971_MOESM1_ESM.docx]
