# Supplementary material for: Pro-angiognetic and pro-osteogenic effects of human umbilical cord mesenchymal stem cell-derived exosomal miR-21-5p in osteonecrosis of the femoral head
Source: Cell Death Discov. 2022 Apr 25;8:226. doi: 10.1038/s41420-022-00971-0 (PMC9039080; doi:10.1038/s41420-022-00971-0)
Supplement: Supplementary file 2 — Supplementary materials (supplementary figures and tables) [file 41420_2022_971_MOESM2_ESM.docx]

**Supplementary Table 1** Sequences for shRNAs

| shRNA | Sequence (5'-3') |
| --- | --- |
| sh-NC | GATGAAGAGCACCAACTC |
| shSOX5-1 | GAGCTGATCATATCCTGCATGTCAG |
| shSOX5-2 | GAGGGTCGTGTTCAATCTTAATTAA |

**Notes:** sh, shRNA; NC, negative control; SOX5, SRY-box transcription factor 5

**Supplementary Table 2** Primer sequences for RT-qPCR

| Genes | Sequence (5'-3') |
| --- | --- |
| hsa-miR-21-5p | F: TAGCTTATCAGACTGATGTTGA |
|  | R: Universal reverse primer of the kit |
| SOX5 | F: CAGCCAGAGTTAGCACAATAGG |
|  | R: CTGTTGTTCCCGTCGGAGTT |
| EZH2 | F: TTTCCAACACAAGTCATCCC |
|  | R: ATAAACCCACATTCTCTATCCC |
| U6 | F: CGCTTCACGAATTTGCGTGTCAT |
|  | R: Universal reverse primer of the kit |
| GAPDH | F: AGAAGGCTGGGGCTCATTTG |
|  | R: AGGGGCCATCCACAGTCTTC |
| syn-cel-miR-39-3p | F: TCACCGGGTGTAAATCAGCTTG |
|  | R:Universal reverse primer of the kit |
| EZH2 (rat) | F: TCTCACCAGCTGCAAAGTGT |
|  | R: AGAGGAGTTGTGTTTTCCCACT |
| SOX5 (rat) | F: GGTCGACACCTTGAAGCAGA |
|  | R: GGAGTTGCTCCCGTAGACTG |

**Notes:** RT-qPCR, reverse transcription quantitative polymerase chain reaction; F, forward; R, reverse; miR, microRNA; SOX5, SRY-box transcription factor 5; EZH2, enhancer of zeste homologue 2; GAPDH, glyceraldehyde-3-phosphate dehydrogenase.

**
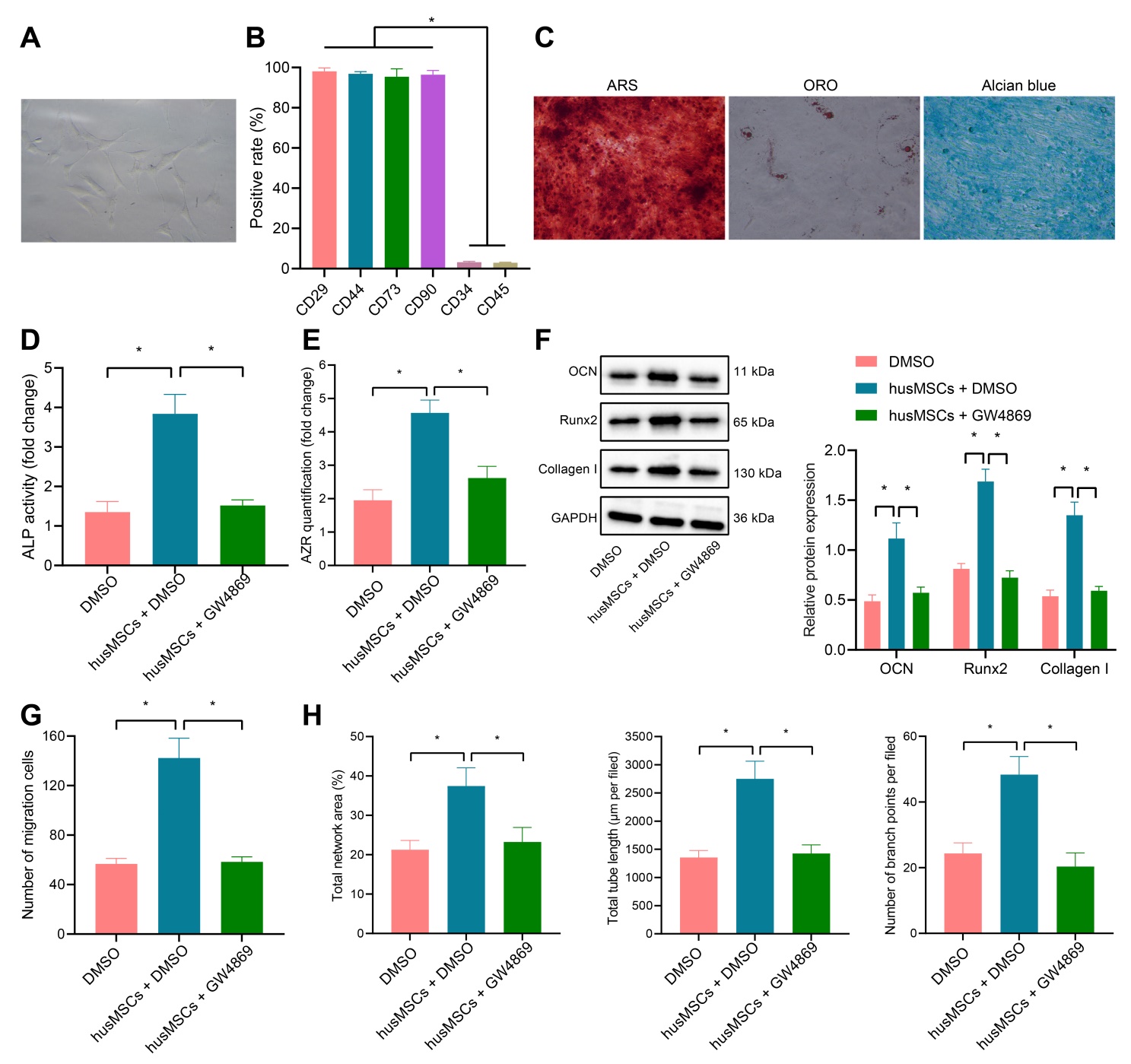
**

**Supplementary Fig. 1** hucMSC-Exos augment HUVEC angiogenesis and hFOB1.19 cell osteogenesis. A: The morphology of hucMSCs observed with an inverted microscope; B: The expression of positive markers (CD29, CD44, CD73 and CD90) and negative markers (CD34 and CD45) of hucMSCs analyzed by flow cytometry; C: Effect of hucMSCs on ssteogenic, adipogenic, and chondrogenic capacity analyzed with Alizarin red S staining, oil red O staining and Alcian blue staining; D: Quantitative analysis of ALP activity of hFOB1.19 cells; E: Quantitative analysis of calcium deposition of hFOB1.19 cells by alizarin red S staining; F: Immunoblotting analysis of the expression of osteogenesis-related markers (OCN, Runx2 and Collagen I) in hFOB1.19 cells; G: The migration of HUVECs determined with Transwell assay; H: The tube-forming ability of HUVECs with total network area, total length and number of branch points determined with capillary-like tube formation assay. Cell experiment was repeated 3 times independently. * *p* < 0.05.

**
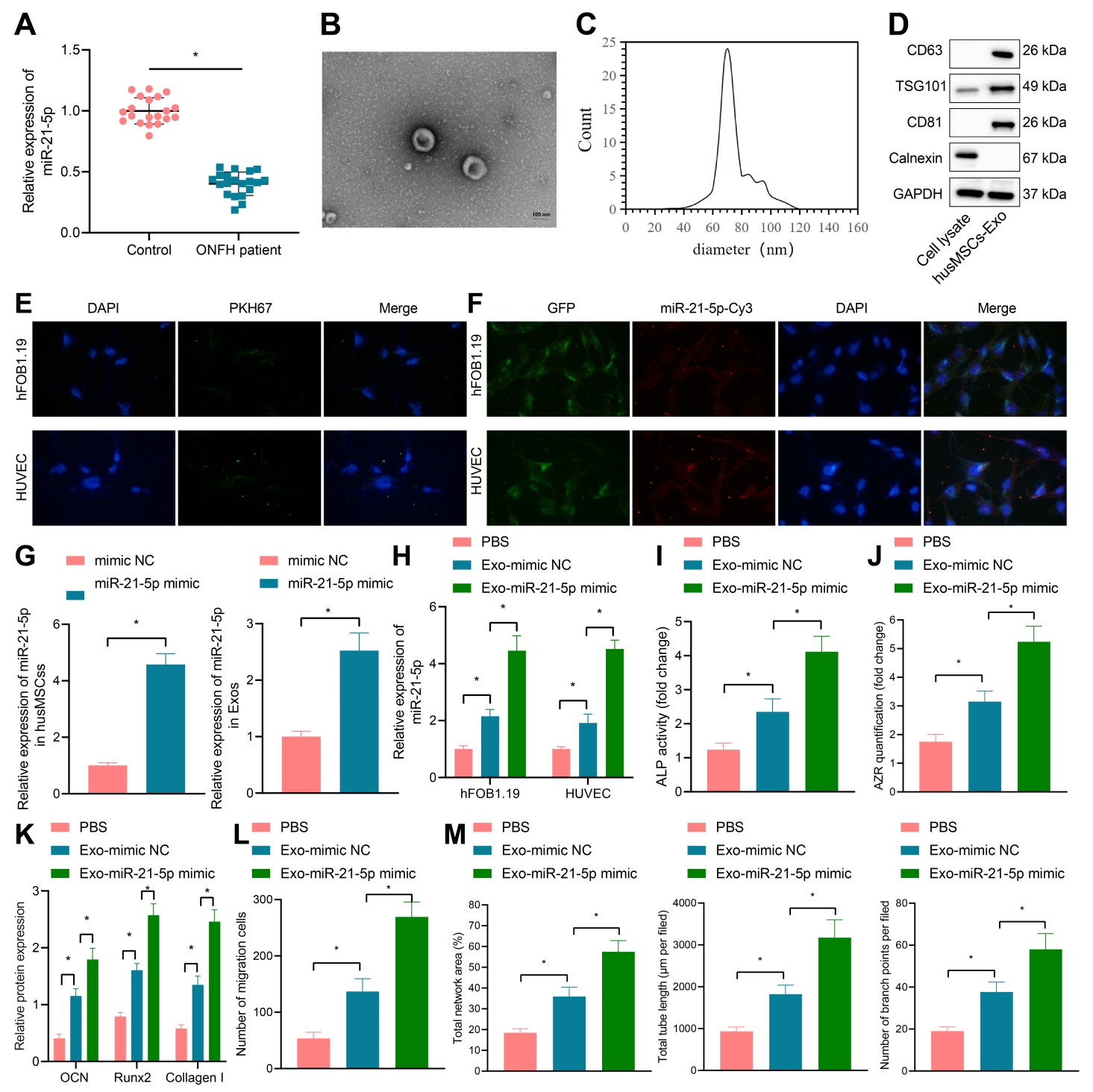
**

**Supplementary Fig. 2** hucMSC-Exos augment HUVEC angiogenesis and hFOB1.19 cell osteogenesis by delivering miR-21-5p. A: The expression of miR-21-5p in clinical tissues of ONFH patients and patients with femoral neck fracture (normal control) determined with RT-qPCR; B: The isolated Exos observed under a TEM; C: Dynamic light scattering analysis of the diameter of hucMSC-Exos; The abscissa represents the diameter of hucMSC-Exos and the ordinate represents the count. D: Immunoblotting analysis of the expression of Exo surface markers (CD63, CD81 and TSG101) and endoplasmic reticulum marker protein Calnexin; E: The uptake of hucMSC-Exos by hFOB1.19 cells and HUVECs observed under an inverted fluorescence microscope; F: hFOB1.19 cells and HUVECs observed under an inverted fluorescence microscope after transfection with miR-21-5p-Cy3 and pCDNA3.1-GFP respectively; G: The expression of miR-21-5p in hucMSCs and hucMSC-Exos determined with RT-qPCR; H: The expression of miR-21-5p in hFOB1.19 cells and HUVECs co-cultured with Exo-miR-21-5p mimic determined with RT-qPCR. I: Quantitative analysis of ALP activity of hFOB1.19 cells co-cultured with Exo-miR-21-5p mimic; J: Quantitative analysis of calcium deposition of hFOB1.19 cells co-cultured with Exo-miR-21-5p mimic by alizarin red S staining; K: Immunoblotting analysis of the expression of osteogenesis-related markers (OCN, Runx2 and Collagen I) in hFOB1.19 cells co-cultured with Exo-miR-21-5p mimic; L: The migration of HUVECs co-cultured with Exo-miR-21-5p mimic determined with Transwell assay; M: The tube-forming ability of HUVECs co-cultured with Exo-miR-21-5p mimic with total network area, total length and number of branch points determined with capillary-like tube formation assay. Cell experiment was repeated 3 times independently. * *p* < 0.05.

**
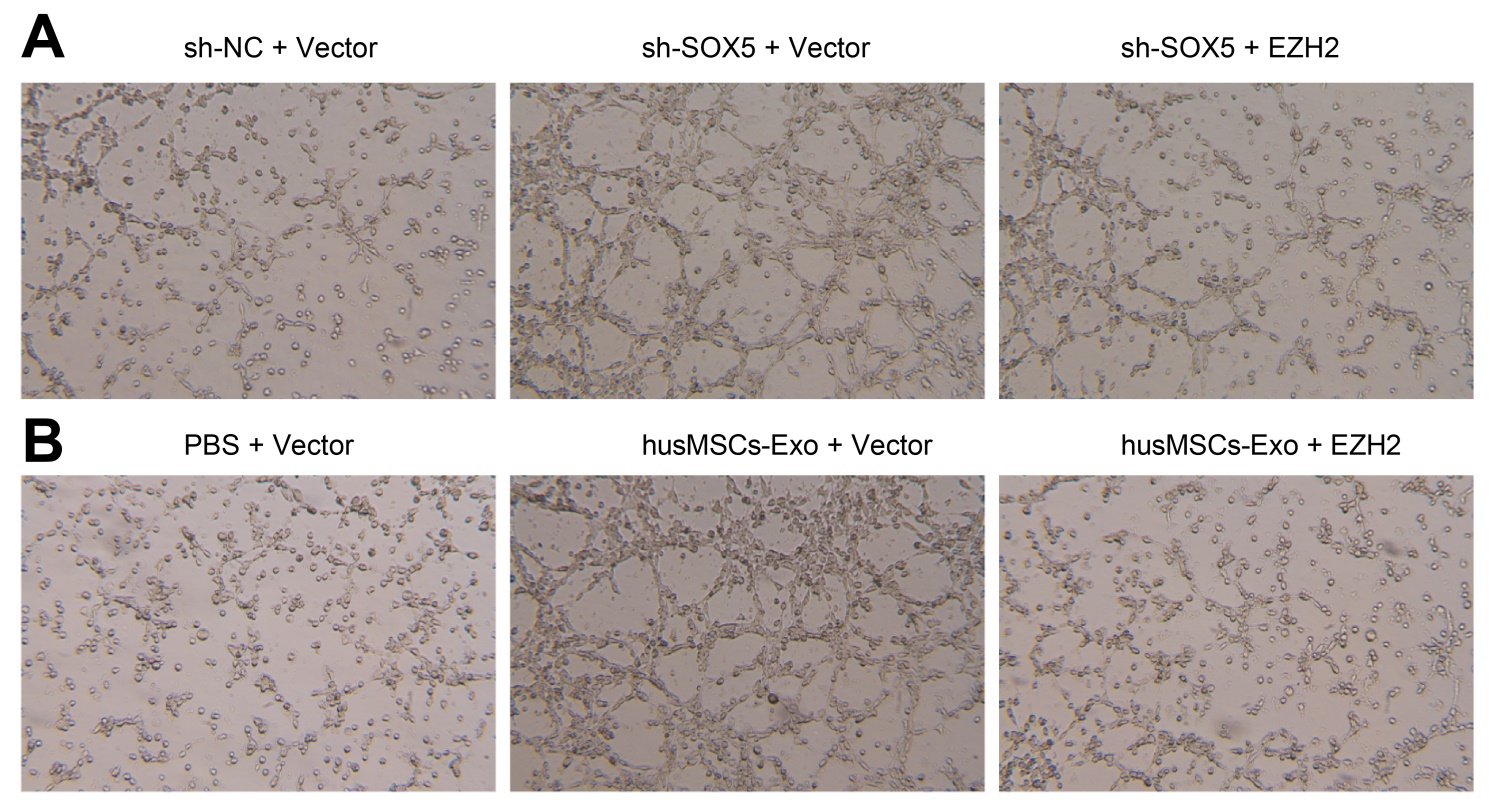
**

**Supplementary Fig. 3** Representative images of capillary-like tube formation assays for Fig. 3G (A),and Fig. 4G (B).
